# The use of blood test trends in cancer detection: a scoping review

**DOI:** 10.1186/s41512-026-00236-9

**Published:** 2026-09-14

**Authors:** Sufen Zhu, Claire Friedemann Smith, Kiana K. Collins, Sheba E. Ziyenge, Jacqueline Murphy, Isabella de Vere Hunt, Zach Brubert, Nia Roberts, Eva J.A. Morris, Richard Hobbs, Brian D. Nicholson, Pradeep S. Virdee

**Affiliations:** 1 Nuffield Department of Primary Care Health Sciences, University of Oxford, Oxford, OX2 6GG UK; 2 Bodleian Health Care Libraries, University of Oxford, Oxford, OX16 9AL UK; 3 Nuffield Department of Population Health, University of Oxford, Oxford, OX3 7LF UK

**Keywords:** Blood test trend, Cancer detection, Repeated measurement, Scoping review

## Abstract

**Background:**

Monitoring trends in repeated blood tests might offer opportunities to improve cancer detection. This review aimed to systematically map the existing literature on blood test trends in cancer detection, characterise how these trends have been used in cancer detection approaches, and identify research gaps.

**Methods:**

MEDLINE, Embase, ProQuest Dissertations & Theses Global, and Overton were searched from inception to April 2025 to identify published and unpublished studies investigating pre-diagnostic blood test trends for cancer detection. Screening was performed by four reviewers. Data extraction was conducted independently by two reviewers, with discrepancies resolved through consensus. Data were narratively synthesised to summarise study and patient characteristics, and the blood tests and cancers investigated. Major themes were identified to categorise key research areas and describe how blood test trends have been used in cancer detection.

**Results:**

Ninety studies were included. Colorectal and pancreatic cancers were the most commonly studied cancers, with haemoglobin and HbA1c the most frequently investigated blood tests for each, respectively. Studies primarily focused on either the association between blood test trends and cancer (*n* = 64) or on prediction models incorporating blood test trends to predict cancer risk (*n* = 26). Additional thematic areas covered by the included studies were: combining trends with other risk factors for cancer (*n* = 62), implication for practice (*n* = 58), blood tests trends versus abnormality (*n* = 40), combination of trends in different blood tests (*n* = 39), and bias due to informative observation (*n* = 12). However, evidence for many other cancer types was scarce, and important gaps remain in understanding how blood test trends can be further used for cancer detection, including their predictive value and implementation strategies.

**Conclusion:**

Evidence to date indicates that blood test trends have been increasingly studied in the context of cancer detection, although knowledge remains fragmented and largely exploratory. Future research should expand to under-studied cancers and examine these trends across a broader range of contexts to better understand their role in cancer detection.

**Registration:**

Open Science Framework https://osf.io/ue8s4.

**Supplementary Information:**

The online version contains supplementary material available at https://doi.org/10.1186/s41512-026-00236-9.

## Introduction

Cancer is a leading cause of death globally, accounting for one in six deaths worldwide and more than one in four deaths in the UK in 2020 [1, 2]. When diagnosed at an early stage, the chances of successful treatment and survival are substantially higher [3]. However, around half of all cancer cases (45.5%) are diagnosed at advanced stages (stage 3 and 4) in the UK [4]. Most cancers are commonly diagnosed after symptomatic presentation to primary care [5]. General practitioners (GPs) are therefore key to enhancing early diagnosis rates but they face multiple challenges in identifying patients at high risk of cancer, including the low prevalence of many cancers, the non-specific nature of cancer symptoms, and the presence of comorbidities [6]. Existing electronic risk assessment tools, which mainly include information on patient data (e.g., family history and BMI) and symptoms, have been developed to help GPs to facilitate cancer referral [7, 8]. However, their usefulness in predicting harder-to-suspect cancers, which usually present with non-specific symptoms, such as myeloma and pancreatic cancer, remains uncertain [6]. Researchers have proposed that one approach to achieve greater improvements in early detection may rely on optimizing the use of existing tests (e.g., blood tests) and identifying accurate biomarkers [6, 9].

Abnormalities identified through standard commonly available blood tests in primary care settings (e.g. full blood counts and liver function tests) play a crucial part in detecting and risk stratifying for possible cancer in symptomatic patients [10]. However, the risk of individual cancers associated with abnormality in most of the blood tests is too low to justify an urgent cancer investigation, apart from anaemia for colorectal cancer and jaundice for pancreatic cancer [11]. When combining all cancers, low albumin, raised platelets, raised calcium, and raised inflammatory markers are the blood test abnormalities that could prompt urgent investigation, as they increase the risk of cancer above the 3% referral threshold recommend by the National Institute for Health and Care Excellence (NICE) [10]. Even though these abnormalities provide GPs with an indication of potential underlying cancer, they leave uncertainty regarding the specific cancer(s) that warrant investigation. Smarter use of standard and non-specific blood tests may offer improved risk stratification for specific cancer sites [10].

Blood test trends refer to patterns of change in quantitative repeated measurements of the same blood test within an individual over time, rather than interpretation based on a single measurement or binary abnormality threshold. Monitoring these trends may better capture within-person changes, be more robust to measurement error, and provide additional information beyond single tests [12, 13]. Incorporating this longitudinal patient information may help to improve the overall predictive accuracy [14, 15]. For instance, a patient with a haemoglobin level within the low-normal range may not be considered to be at high risk when interpreting the result in isolation with a binary threshold for abnormality. If this low-normal result follows years of consistently high-normal results, it could signify an opportunity for cancer investigation. Incorporating trends in repeated blood tests may, therefore, offer enhanced predictive advantage and contribute to more individualised risk stratification for cancer [16].

A recent systematic review collated published studies to assess whether blood test trends are associated with underlying cancer [17]. The scope of the review was limited, with the broader context of literature regarding blood test trends for cancer detection not being explored. For example, it is unclear whether there is any evidence on whether trends can predate symptom presentation or blood test abnormality (and by how long), the cost-effectiveness of repeat blood testing, or how they could be appropriately implemented into clinical practice and guidelines to inform patient selection for referral. This review aimed to systematically map the existing literature on the use of blood test trends for cancer detection, characterise how these trends have been applied, and identify research gaps.

## Methods

This review was conducted in accordance with JBI methodology for scoping reviews and followed the Preferred Reporting Items for Systematic Reviews and Meta-Analysis extension for Scoping Reviews (PRISMA-ScR) [18].

### Search strategy

With the support of an experienced librarian (NR), we conducted a systematic search to locate both published and unpublished studies, using MEDLINE (Ovid), Embase (Ovid), ProQuest Dissertations & Theses Global and Overton (see Appendix 1 for an example of search strategies in MEDLINE (Ovid)). The reference list of all included studies was manually inspected to locate further relevant studies. Studies published from inception until 03 April 2025 were included, with no language restriction applied.

### Study selection

The eligibility criteria were structured following the population, concept and context categories for scoping review. For population, we included studies with patients of any demographic. To meet the concept criteria, studies needed to report change over time in repeated blood tests, which included at least one blood test commonly available in primary care (Table 1). We included studies that investigated diagnosis of any type of cancer. In addition, eligible studies had to investigate trends in blood test use before and/or during cancer diagnosis. Those investigating blood tests use after cancer diagnosis, such as those used for prognosis prediction or treatment monitoring, were excluded. For context, studies from all settings and geographic regions were considered eligible. This scoping review included studies of any study design, including but not limited to primary research, literature reviews, commentaries, editorials and letters. Grey literature, such as theses, dissertations, conference proceeding and posters, were also included. Other non-research evidence or knowledge, such as news articles, websites or guidelines, were excluded. In cases where the same data are reported in multiple publications, the primary article or the one with the most comprehensive data for exaction and analysis was prioritized.

Following the search, all identified records were collated and uploaded into EndNote 21 (Clarivate Analytics, PA, USA) and duplicates were removed. Multiple reviewers (SZ, CFS, KKC and PSV) screened the titles and abstracts against the inclusion criteria for the review in Endnote 21 and/or Rayyan. Subsequently, we performed full-text screening on studies considered potentially relevant at the prior title and abstract screening stage. Reasons for exclusion of studies at full text screening were recorded and reported.

**Table 1:** Blood tests under investigation

Blood test	Blood level
Full Blood Count	Red blood cell count, haemoglobin, haematocrit, mean cell volume, mean cell haemoglobin, mean cell haemoglobin concentration, red blood cell distribution width, platelet count, mean platelet volume, white blood cell count, basophil count, eosinophil count, lymphocyte count, monocyte count, neutrophil count, basophil %, eosinophil %, lymphocyte %, monocyte %, neutrophil %
Liver Function Tests	Alanine aminotransaminase, albumin, alkaline phosphatase, aspartate transaminase, bilirubin
Renal Function	Sodium, potassium, creatinine, urea
Inflammatory Markers	C-reactive protein, erythrocyte sedimentation rate, plasma viscosity
Other tests	Amylase, HBA1c, calcium, calcium adjusted, total protein, blood glucose, fasting glucose, thyroid stimulating hormone

### Data extraction

Two reviewers extracted data from eligible studies independently, using an extraction form developed by the research team. The form was piloted on a random sample of 10% of the included studies, and was modified and revised as necessary during the process of extracting data from each included study. Data extracted included information on study characteristics (e.g., study design and setting), participants (e.g., age and sex), blood test (e.g., type of tests and definition of blood test trend), cancer (e.g., type of cancer and time of diagnosis), utilization of blood test trends for cancer, analytical methods used related to the blood test trends and cancer, and any implications of blood test trend for clinical practice. Discrepancies between the two reviewers were discussed until agreement was reached or by consultation of a third reviewer. Authors of papers were contacted, where required, to request missing data.

### Data analysis and reporting

Extracted data on study characteristics, participants, blood tests, and cancer were synthesised narratively. To visualise the scope of the literature, a heatmap was created to display the frequency with which specific blood tests were investigated for different cancer types. Cancer type were grouped based on medical specialty; cancers investigated in less than three studied were combined into the “other” category. To characterise the use of blood test trends in cancer detection, we identified major themes to categorise the key areas covered by different studies. Major themes were developed and iteratively refined by repeated discussion amongst three reviewers (SZ, PSV and BDN). For each theme, we recorded the number of studies addressing it to enable a structured overview of the breadth and distribution of research focus in this field. Under each major theme, we captured different methodological approaches to investigate blood test trend and cancer.

## Results

**Fig. 1:**
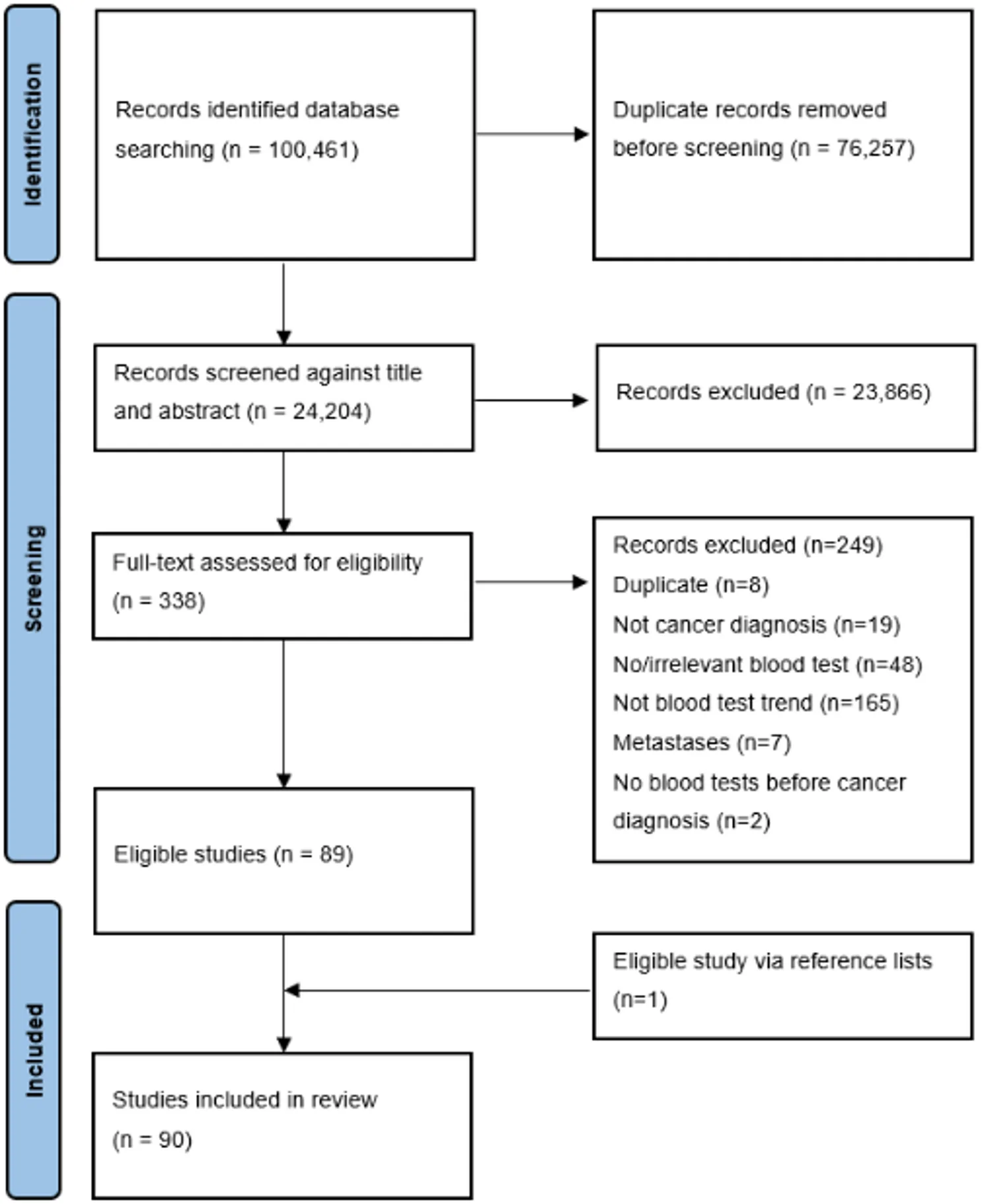
PRISMA diagram

A total of 24,204 records were identified through the literature search after removal of duplicates (Fig. 1). Following title, abstract, and full-text screening, 89 studies met the inclusion criteria. An additional eligible study was identified through reference list screening of the included studies [19], bringing the total number of included studies to 90. Publication dates of the included studies ranged from 1984 to 2025 with the majority of studies published recently (2004 or earlier, *n* = 4; 2005–2009, *n* = 2; 2010–2014, *n* = 9; 2015–2019, *n* = 31; 2020–2025, *n* = 44). Most of the included studies were conducted in high-income countries (*n* = 85), including the US (*n* = 36) and the UK (*n* = 19). Cohort is the most common study design (*n* = 45), followed by case-control (*n* = 33), case report (*n* = 8), case study (*n* = 3), protocol (*n* = 1) and review (*n* = 1).

**Fig. 2:**
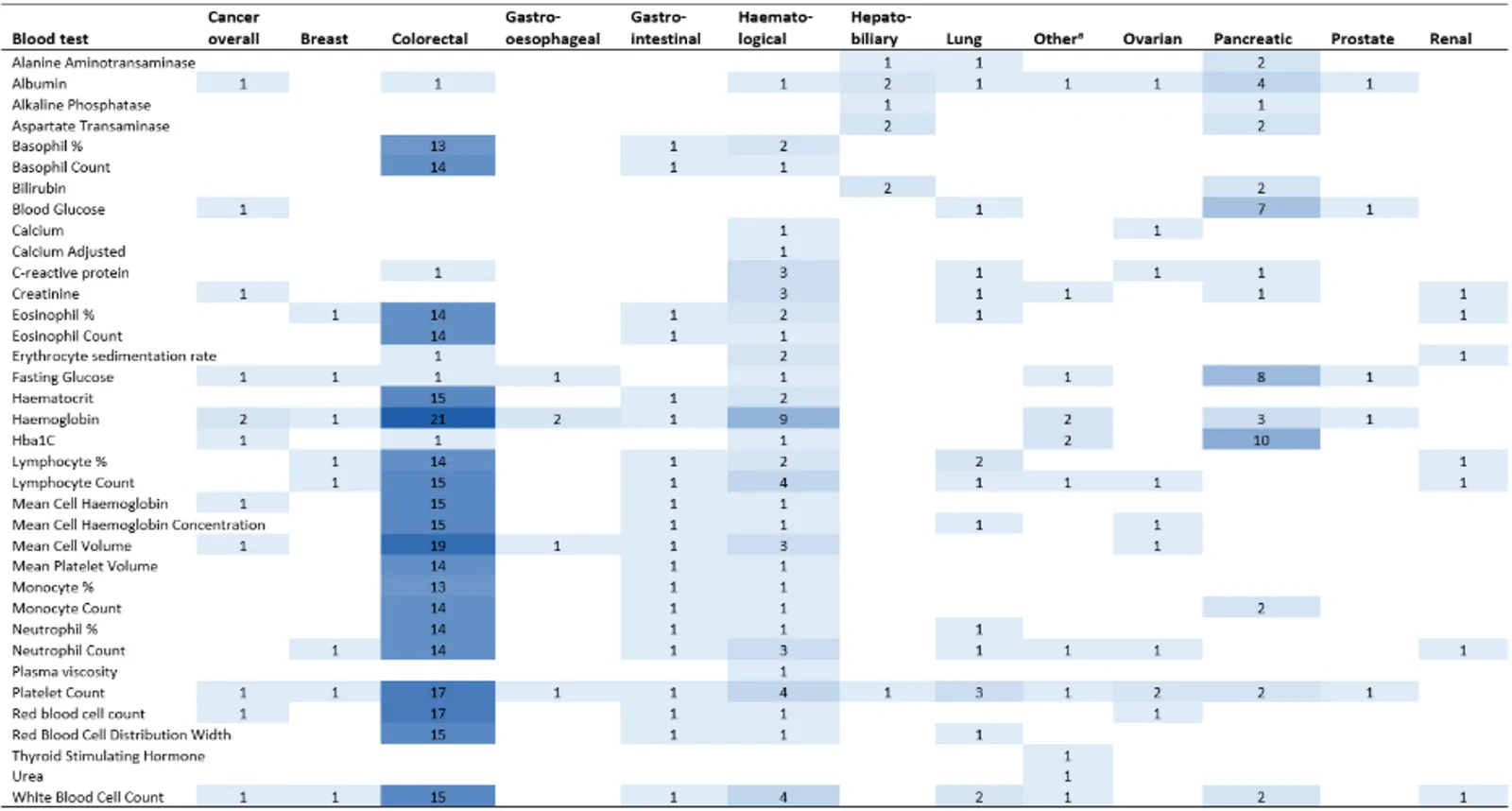
Heatmap showing the number of studies by cancer type and blood test. ^a^ Other cancer included small intestinal, thyroid, neuroendocrine, skin and glioma cancer

The number of studies by type of cancer and blood test is shown in Fig. 2. Colorectal cancer (*n* = 26) and pancreatic cancer (*n* = 24) were the most commonly studied cancer types, followed by haematological cancers (*n* = 16) and lung cancer (*n* = 7). Haemoglobin was the most frequently investigated blood test, examined in 34 studies, followed by platelet count (*n* = 28). The number of studies for each blood test and cancer type is presented in Table 2. Among studies investigating colorectal cancer, haemoglobin (*n* = 21) and mean cell volume (*n* = 19) were the most frequently assessed tests, whereas HbA1c (*n* = 10) and fasting glucose (*n* = 8) were the most common in studies focusing on pancreatic cancer. Detailed study characteristics are shown in Appendix 2.

**Fig. 3:**
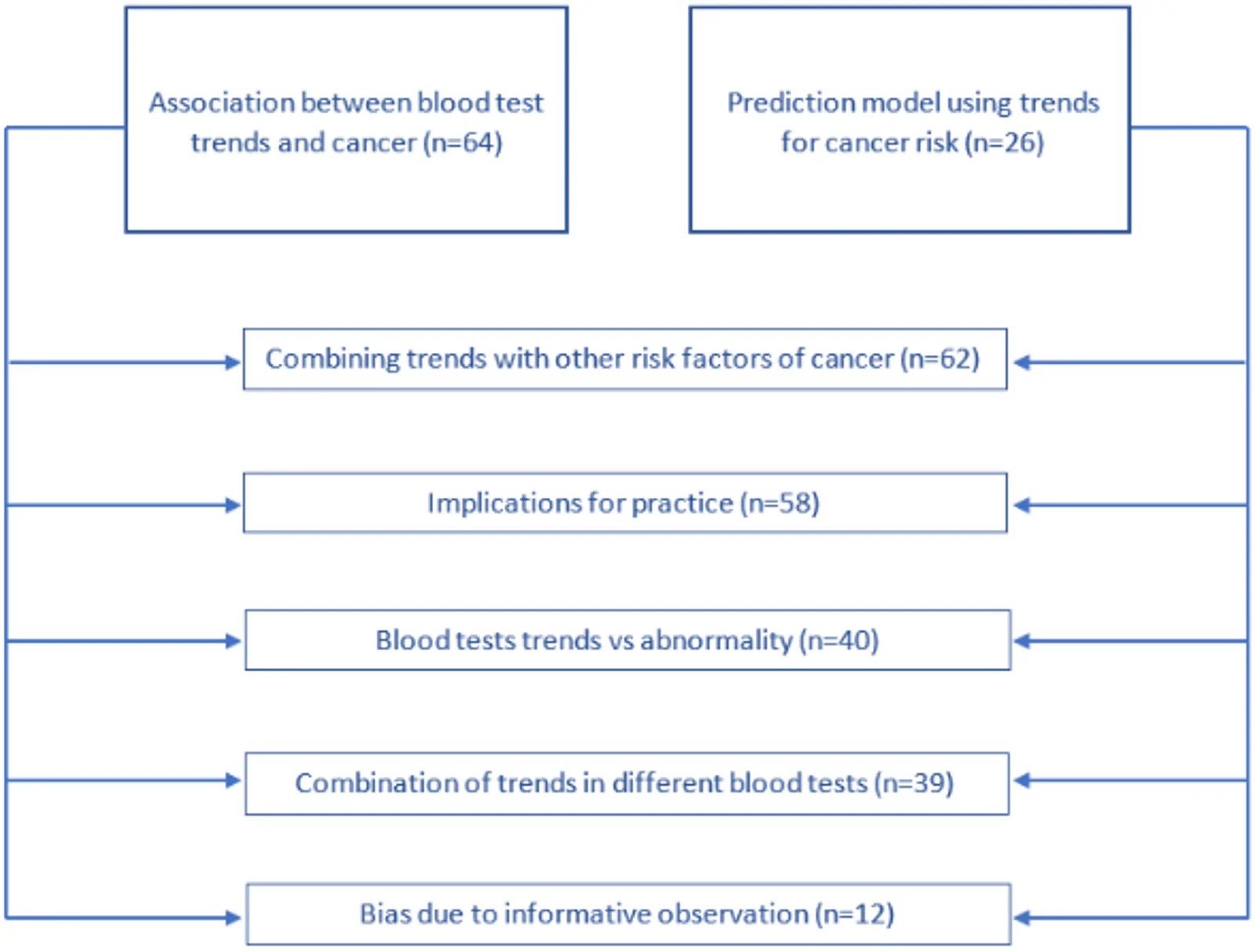
Common themes identified on blood test trends for cancer detection

We identified and categorised the key terms into seven common themes. While the studies primarily focused on either the association between blood test trends and cancer (*n* = 64) [20–83] or on prediction models incorporating blood test trends to derive cancer risk (*n* = 26) [19, 84–108], many addressed additional thematic areas in their studies (Fig. 3). A summary of current literature and research gaps is shown in Table 2.

### Association between blood test trends and cancer

Most included studies (*n* = 64) [20–83] examined the association between blood test trends and cancer, employing a variety of methodological approaches to investigate this relationship. Approaches included descriptive reporting of blood test results at different time points (*n* = 22) [20, 23, 24, 27, 35, 40–44, 46, 49, 50, 53, 54, 57, 61, 66, 72, 76, 82, 83], and regression-based methods to model temporal changes in blood test values (*n* = 20) [22, 25, 30, 31, 33, 38, 39, 43, 44, 48, 51, 52, 58, 63–65, 71, 74, 77, 81], most commonly using linear regression. Several studies used statistical tests to compare blood test values at different time points between patients diagnosed with cancer and those without (*n* = 13) [25, 30, 36, 39, 58–60, 63, 64, 70, 71, 73, 78], while others explored associations between cancer and quantified changes in blood tests, such as absolute or relative changes or the slope of change over time (*n* = 12) [26, 33, 37, 42, 47, 48, 65, 67, 69, 75, 79, 82]. Ten studies examined how measures of association, such as risk ratios and hazard ratios, between blood test results and subsequent cancer diagnosis varied across different time intervals before diagnosis [21, 29, 31, 44, 51, 55, 56, 62, 68, 77]. Seven studies categorised the trajectories of blood test results, for example using group-based trajectory models, and analysed the association between the categorised trend and cancer [28, 32, 34, 45, 47, 56, 80]. Two studies focused on modelling individual-level blood test trajectories, using multivariable joint modelling and linear mixed-effect model [81, 83].

**Table 2:** Summary of current literature and research gaps

Domains	Current literature	Research gaps
Association between blood test trends and cancer	Descriptive reporting; regression models of change in blood tests; comparison of blood test values at different time points; absolute or relative change in blood test values; change of association (e.g., change of odds ratio) over time; categorised group-level trajectories; individual-level trajectories	No assessment of how the number or timing of repeated tests influences associations, or whether different analytical methods affect results; limited use of longitudinal methods designed for repeated measurements; limited studies on individual-level blood test trends
Prediction model using trends for cancer risk	Model development; comparison of models developed using different methods; model validation	No comparison between trend-based models and models using single-test abnormalities; limited studies on broader types of cancer; limited use of individual-level dynamics of blood tests; limited external validation
Combining trends with other risk factors of cancer	Adjustment (most commonly for age, sex and BMI); stratification ((most commonly for sex and diabetes status); descriptive reporting; comparison of time sequences	Limited integration of symptom information or other diagnostic tests
Implications for practice	Trends as indicators of undiagnosed cancer; general statement on the potential of trends; detailed suggestions on practical considerations; importance of repeated measurements	Limited actionable implications; no recommendations for incorporating trends into clinical guidelines for general practitioners; no evidence of actual implementation of trend-based models in practice
Blood tests trends vs. abnormality	Graphical presentation; potential of trend to pre-date abnormality; descriptive reporting; proportion of abnormality over time; comparison of model performance	Limited studies on diagnostic utility or predictive performance of trends compared to abnormality
Combination of trends in different blood tests	Descriptive reporting of the concurrent changes; inclusion in modelling; changes in the ratios of different tests; combined effect of trajectory groups	No assessment of predictive performance of multiple trends versus individual trends; limited focus on tests beyond full blood count; limited evidence on correlations between different blood test trends
Bias due to informative observation	Acknowledgement as potential limitation; comparison of frequency of blood tests among different groups	No proposed or applied method to address or mitigate this bias
Overall	Focus on certain types of cancer and blood tests, and the identified 7 major themes	Limited research on many other types of cancers and blood tests; no evidence on broader use of trends, e.g., perspectives of patients and clinicians, acceptability and feasibility of using blood test trends in real-world care, cost-effectiveness of repeat blood testing and trend, and implementation

Studies examined blood test trends at different analytical levels. Trends were analysed at the individual level in 26 studies [20, 24, 27, 28, 32, 34, 35, 41, 45–47, 50, 53, 54, 56, 57, 61, 65, 66, 72, 76, 79–83], typically modelled within-person changes in test results over time, with only one study examining the correlation between repeated measurement. Thirty-three studies assessed population-level trends [22, 23, 25, 26, 30, 31, 33, 36–40, 42–44, 48, 49, 51, 52, 58–60, 63, 64, 67, 69–71, 73–75, 77, 78], often using aggregated data or comparing average test values across groups. Five studies did not examine temporal trends directly but instead focused solely on measuring associations between static test results and cancer incidence at different time points [21, 29, 55, 62, 68]. No study assessed how the number of or timing between repeated tests influences the association. No study compared whether employing different methods to analyse repeated measures influenced any association.

Across these studies, blood test trends were often reported to change in the period leading up to cancer diagnosis, with gradual shifts observed in biomarkers such as haemoglobin and glucose. However, findings were inconsistent in terms of timing and magnitude.

### Prediction model using trends for cancer risk

Twenty-six studies focused on the development or evaluation of cancer risk prediction models that incorporated blood test trends [19, 84–108]. Five studies developed 8 models in total [93, 99, 102, 104, 106], aimed at predicting the risk of specific cancers: colorectal cancer (*n* = 2), gastrointestinal cancer (*n* = 1), lung cancer (*n* = 1), and pancreatic cancer (*n* = 1). A range of modelling approaches was used across studies, including machine learning (*n* = 3), logistic regression (*n* = 3), and multivariate joint modelling (*n* = 2). Machine learning methods comprised XGBoost (*n* = 1), random forest (*n* = 1), and a combination of gradient boosting with random forest (*n* = 1). For logistic regression models, two studies incorporated changes in values or categories from repeated blood tests as single predictors [102, 104], while one study did not clearly describe how trends were included [93]. One study applied multivariate joint modelling to account for variable test frequency and timing in repeated measures data for both models it developed [106]. Two studies compared the performance of prediction models developed using different analytical approaches, namely logistic regression and machine learning [93, 102]. One study specifically evaluated the comparative performance of models based on single time point blood test results versus longitudinal measurements [102]. The point estimates of the Area Under the Curve (AUC) values of these models ranged from 0.71 to 0.87. External validation was conducted in 14 studies [84–86, 88–90, 92, 95–98, 100, 103, 106], evaluating two established models: ColonFlag (with AUC values ranging from 0.74 to 0.84) (*n* = 10) and ENDPAC (with AUC values ranging from 0.69 to 0.75) (*n* = 4). The feasibility of implementing a cancer risk prediction model in routine clinical practice was the focus of one proposed (protocol) study [91], while another study synthesised existing evidence on ColonFlag and assessed its overall efficacy [101]. One study examined whether the ColonFlag algorithm could identify undiagnosed colorectal cancer prior to symptom onset and assessed its ability to detect increased risk even when full blood count (FBC) test results were within normal ranges [19]. No study compared the risk of their trend-based prediction model to the risk of blood test abnormality on single tests.

In two studies [87, 108], blood test trends were initially considered as candidate covariates during model development but were excluded from the final models due to lack of statistical significance. Three additional studies considered repeated blood test measurements in their models [94, 105, 107], with each test being a separate variable, so did not explicitly model temporal trends.

### Combining trends with other risk factors of cancer

There were 62 studies that combined blood test trends with other cancer risk factors in their investigations [19–21, 24, 26–29, 32–35, 37, 39, 41–44, 49, 51–53, 55, 56, 58, 61, 62, 64–66, 68, 69, 71–73, 75–77, 79, 81–86, 88–93, 95–104, 106]. Most of these studies incorporated additional risk factors through adjustment in analyses or prediction models (*n* = 39), most commonly age, sex and BMI (including 16 external validation studies and studies exploring prediction models in wider contexts that involved existing prediction models, either validating them or exploring further aspects of these models) [19, 21, 26, 28, 29, 32, 33, 37, 51, 55, 56, 62, 65, 68, 75, 79, 81–86, 88–93, 95–104, 106]. Subgroup or stratified analyses of blood test trends were conducted in 26 studies to assess the influence of other risk factors, most frequently by sex (*n* = 13) and diabetic status (*n* = 7), followed by age (*n* = 3), BMI (*n* = 2), ethnicity (*n* = 2), and clinical conditions (*n* = 1) [32, 39, 42–44, 49, 51, 52, 58, 64, 66, 68, 69, 71, 73, 77, 81–83, 86, 90, 95, 96, 102, 103, 106]. A small number of studies (*n* = 9) [20, 24, 27, 35, 41, 53, 61, 72, 76], primarily case reports, descriptively illustrated concurrent changes in blood test results alongside any symptom presentation or various comorbidities, including jaundice and diabetes, reflecting typical clinical practice in which doctors simultaneously consider these factors for diagnosis, and some cases, symptoms directly prompted blood testing. One study investigated whether blood test trends could detect undiagnosed cancer prior to the onset of colorectal cancer symptoms, such as rectal bleeding and appetite loss [19]. Another study combined trajectories from one blood test of interests with a different blood test outside the scope of this review, creating combined trajectory groups associated with varying levels of cancer risk [34].

### Implications for practice

Implementation of blood test trends in clinical practice was discussed in 58 studies [19, 23, 25, 28, 30, 31, 34, 36, 38, 39, 41–44, 47–55, 57–59, 61, 66, 69, 70, 72, 73, 75, 76, 78, 79, 81–86, 88, 90–93, 95–104, 106]. Most of them (*n* = 19) highlighted how particular trend patterns could act as signals warranting consideration of cancer or as markers for risk stratification [23, 25, 34, 38, 47, 48, 52–55, 59, 61, 66, 72, 73, 75, 76, 78, 82]. Some studies (*n* = 19) offered only broad statements, suggesting that trend-based models might, or might not, potentially be used in clinical decision-making, population screening, or within health systems, but without describing mechanisms for implementation [28, 36, 39, 44, 49, 51, 57, 69, 70, 79, 83, 84, 88, 93, 96–98, 100, 103, 104]. A smaller group of studies (*n* = 18) offered more detailed and explicit suggestions, outlining practical considerations such as integration with electronic health records, the impact of risk thresholds on sensitivity and specificity, or how trends might complement existing referral pathways and screening frameworks [19, 30, 31, 41–43, 58, 81, 85, 86, 90, 92, 95, 99, 101–103, 106]. Three studies emphasized the importance of repeated measurements [50, 52, 57], while one study specifically proposed testing the feasibility of implementing a trend-based model in a primary care setting [91]. No study provided recommendations for incorporating trends into clinical guidelines for general practitioners, and none reported actual implementation of trend-based models in clinical practice.

### Blood tests trends vs. abnormality

Studies examining the relationship between blood test trends and abnormality with cancer highlighted different ways of interpreting changes over time relative to conventional threshold-based approaches. Many studies (*n* = 22) graphically illustrated changes in blood test results over time that were often confined with the normal range without performing inferential analyses comparing trends to thresholds of abnormality [25, 26, 31–34, 38–40, 42, 49–51, 58, 59, 63, 66, 71, 74, 77, 81, 82]. Three studies explicitly highlighted the potential of trend to pre-date abnormality [19, 44, 52]. Eight case reports described individual patients’ blood test results at multiple time points, with abnormality assessed on a case-by-case basis [20, 24, 27, 35, 50, 53, 61, 72, 76]. Eight studies reported the proportion of abnormal results prior to or at the time of cancer diagnosis [25, 30, 36, 43, 54, 64, 70, 81], three of which described how this proportion changed over time [30, 64, 81]. Two studies restricted their analyses to patients with abnormal test results [41, 67], while one study examined changes in blood test over time separately for those with normal and abnormal baseline results [82]. One study compared the performance of a prediction model incorporating trends with a model based on single time point values [102].

### Combination of trends in different blood tests

A total of 39 studies investigated combinations of blood test trends for cancer diagnosis. Of these, concurrent changes of multiple blood tests over time were descriptively reported in 20 studies [20, 23, 24, 26, 33, 35, 40, 42, 45, 49, 50, 57, 64, 68, 72–74, 77, 78, 81]. Simultaneous trends in different blood tests were included in models examining associations or predicting cancer risk in 15 studies [19, 37, 48, 75, 84–86, 92, 95, 96, 98–100, 103, 106], while one study combined trend with blood test values measured at a single time point [47]. Four studies examined changes in the ratios between blood tests, such as aspartate aminotransferase to platelet ratio [47, 65, 74, 78], and one study assessed the combined effect of trajectory groups derived from multiple blood tests on cancer risk [28]. Additionally, one study reported the proportion of individuals with an abnormal result in another blood test among those exhibiting a specific blood test trend [54]. Only one study explicitly stated that it assessed the correlation between blood test trends, but the results were not included in the manuscript (but were noted to be available from the authors) [81]. Most studies that combined multiple trends focused primarily on components of the full blood count.

### Bias due to informative observation

Bias due to informative observation was noted in 12 studies [31, 40, 42, 48, 49, 52, 62, 68, 81, 96, 102, 106], stating that generalizability of findings could be affected if the timing or frequency of blood tests is influenced by patients’ underlying health status or clinical outcomes, including work up for cancer diagnosis. The ways in which different patient groups undergo blood testing varied across studies. Huang et al. indicated that patients undergoing multiple blood tests might comprise both healthier individuals adhering closely to routine check-ups and sicker patients requiring frequent monitoring [42]. Conversely, studies by Virdee et al. and Sare-Azodi et al. suggested that patients undergoing blood testing generally appeared to be less healthy compared to those who were not tested [42, 62, 81]. Three studies compared the number or frequency of blood tests between populations and arrived at differing conclusions: one reported differences in the proportions of individuals receiving blood tests between cancer and non-cancer groups [68]; another observed differences between cancer patients and the general population [52]; and the third study found no difference in testing frequency between individuals with and without cancer [31]. Although the studies in this theme commented on bias due to informative observation, no study proposed any effective method or reported attempting to address or mitigate this bias in their longitudinal analyses.

## Discussion

We mapped and reviewed the broad literature on blood test trends for undiagnosed cancer. Colorectal and pancreatic cancers were the most commonly studied cancers, with haemoglobin and HbA1c being the most frequently investigated blood tests for each, respectively. Research on blood test trends for many other types of cancers and blood tests is limited. Studies primarily focused on the association between blood test trend and cancer or derived/validated prediction models incorporating trends. Other critical aspects, including the combined use of blood test trends with other clinical indicators and comparisons between trend-based and abnormality-based approaches, were partially addressed but remain underexplored. Furthermore, the broader role of blood test trends, such as their diagnostic value, the perspectives of patients and clinicians of repeat testing, and the practical implementation considerations of trend analysis in clinical settings, has yet to be clearly investigated.

Substantial methodological heterogeneity was seen across the included studies, which may be attributed to the exploratory nature of the field and the current lack of consensus on optimal strategies for incorporating temporal information into cancer detection. A considerable number of studies employed descriptive analysis, case report, or simplified representations of temporal trends, often reducing changes to comparisons at fixed time points, or using population-level aggregated data or summary measures within simple regression models. Although these approaches might be helpful in highlighting overarching patterns, they are unlikely to adequately capture the complexity and variability of within-person trajectories over time. Advanced longitudinal methods, which are more suitable for explicitly modelling temporal dynamics of repeated measurements, such as machine learning and joint modelling, were underrepresented. Several established methodological approaches exist for harnessing repeated measurements for clinical risk prediction, such as landmarking, time-dependent covariate modelling and two-stage modelling [109], and future research could explore and apply these methods in cancer detection to allow more robust evaluation of the added value of blood test trends. Prediction model development has been limited to a small number of cancer types, with little external validation, raising concerns about generalisability and real-world applicability. Future research should prioritise external validation across diverse populations and expand model development to a broader range of cancers. Critically, studies should directly compare models incorporating blood test trends with those based on standard of care, such as using single time point measurements to define abnormality, to determine whether trends provide meaningful improvements in predictive performance and in practice.

Studies commonly combined blood test trends with demographic and clinical risk factors such as age, sex and comorbidities, while the integration of symptom information and other diagnostic tests remained limited. Symptoms play an important role in cancer detection in primary care, where patients often present with vague or non-specific complaints [110], and tests already embedded in diagnostic pathways may not have the perfect sensitivity and specificity [111]. Integrating these with blood test trends could help determine whether trends improve risk stratification, enhance the accuracy of symptom-based referral pathways, and better prioritise patients for further investigation. Most discussions on blood test trend for cancer detection remained theoretical with limited guidance on how trend-based tools might be integrated into clinical workflows. Bridging the gap between methodological development and clinical application will require interdisciplinary research that moves beyond model performance to evaluate feasibility, integration with clinical systems (e.g., electronic health records), and acceptability to clinicians and patients, alongside engagement with policymakers to support translation into practice.

Current cancer referral guidelines, are largely based on single test abnormality (usually with other criteria), which may fail to capture meaningful clinically relevant trends occurring within the normal range. Although some studies suggest that blood test trends may provide earlier signals of cancer, evidence remains limited and few studies have formally compared trend-based approaches with threshold-based strategies. Future research should compare trend-based, threshold-based, and integrated approaches, using robust statistical methods to evaluate whether incorporating trends improves early detection and risk stratification. Although combination of trends across multiple blood tests has the potential to capture multiple biological signal simultaneously [112], existing evidence remains limited by predominantly descriptive analysis and lack of incremental predictive performance over individual test trajectories. The strong focus on full blood counts likely reflects their widespread availability across healthcare settings, but may constrain the development of more informative models by overlooking other relevant tests, such as liver function, or metabolic markers. Future research should move beyond descriptive approaches and apply multivariate methods across a broader range of tests to determine whether multi-marker trends offer meaningful improvements in cancer detection. Informative observation bias presents a significant methodological concern in studies of blood test trends and cancer risk [113], as blood tests in real-world settings are often driven by emerging symptoms, chronic disease management, or clinician concern, factors that may themselves be associated with underlying cancer risk. As a result, the timing and frequency of testing are not random but are informative: they carry implicit information about a patient’s health status. If not properly accounted for, this can limits the reliability and generalisability of trend-based findings. Future research should adopt appropriate methods, such as inverse probability weighting, joint modelling, or sensitivity analyses ensure valid evaluation of trend-based approaches.

### Strengths and limitations

This review has several strengths. To our knowledge, it is the first to systematically map the broad literature on the wider use of blood test trends for cancer detection, offering a comprehensive overview of current evidence. The inclusion of many databases and both published and grey/unpublished literature enhances the breadth of the review and ensures we maximise the inclusion of relevant literature. Additionally, the use of broad inclusion criteria allowed us to capture a diverse range of study designs, populations, and clinical settings, providing a wide view of how trends have been investigated across contexts.

This review has limitations. Firstly, as this was a scoping review, we did not conduct a systematic quality appraisal of the included studies. We also did not formally assess the level of confidence in each thematic finding. However, we have indicated the number of studies contributing to each theme to provide readers with a sense of the underlying evidence base. Secondly, due to the nature of the existing literature, which is still at an early and exploratory stage, our findings primarily highlight gaps rather than offer insights into the practical implementation of blood test trends, limiting their immediate clinical applicability. The considerable heterogeneity across studies, including varying definitions and applications of trend for cancer, hindered direct comparisons and precluded quantitative synthesis. Although this review included both published and grey literature, our search of published studies was conducted using MEDLINE and Embase, with support from an information specialist. While some relevant published studies may be indexed only in other databases, MEDLINE and Embase are known to have high coverage of biomedical research [114]. Citation searching of included articles further helped reduce the risk of missing key studies.

### Comparison with existing reviews

How to best harness blood test trends to inform our approach to cancer detection is a relatively new and evolving research area despite blood test trends being considered in clinical practice. While routine blood tests are widely ordered in clinical practice, interpretating changes over longitudinal measurements, particularly in the context of early cancer detection, has only recently gained attention in the research literature. Three systematic reviews have examined this area, each with a specific and narrow focus: one explored whether blood test trends are associated with underlying cancer [17], another evaluated the performance of prediction models using trends for cancer [115], and a third focused specifically on the performance of the ColonFlag algorithm across different populations [101]. Although these reviews emphasised the potential value of blood test trends, they were limited in scope and did not capture the full breadth of how trends are currently being studied and applied. This scoping review aimed to comprehensively map the emerging evidence base, providing a wide-angle view of how blood test trends are being used across different research settings and clinical contexts. By including both published and grey literature, including abstracts and theses, and synthesising a broader range of themes, including the integration of trends across multiple blood tests and considerations for clinical implementation, we offer additional insights that the previous reviews did not address. Mapping out this landscape is a crucial step in understanding the current state of the evidence and identifying where further methodological development and clinical research is needed to translate trend-based approaches into clinical practice.

### Research implications

While most studies have focused on a limited set of cancers and blood tests, future work should broaden the scope to include less-studied cancer types and a wider range of routinely used tests. For cancers and blood tests where associations have already been demonstrated and prediction models developed, there is a need to move beyond exploratory analyses by providing external validation across diverse populations and settings and by further exploring their role in cancer detection. This should include formal assessment of diagnostic performance (such as sensitivity, specificity, and lead time), as well as investigations into acceptability and feasibility from patient and clinician perspectives. Cost-effectiveness analyses will be essential to determine their value in resource-limited settings, and implementation studies are needed to establish how trend-based models can be integrated into clinical workflows, decision support systems, and referral pathways. There is also scope for methodological refinement. Limited research has examined how the number and timing of repeated tests influence associations between blood test trends and cancer. Irregular or infrequent testing may obscure meaningful changes, making it unclear how testing schedules should be structured to support timely and accurate diagnosis. Incorporating symptoms and other clinical risk indicators, such as comorbidity, into trend-based prediction models remains largely unexplored but could enhance model performance and clinical relevance. Few studies have compared trend-based approaches with threshold-based or integrated strategies, or with other established diagnostic investigations (e.g., faecal immunochemical testing for bowel cancer), leaving it uncertain whether trends offer added or superior value in real-world diagnostic pathways.

## Conclusion

We mapped a wide range of literature on blood test trends for cancer detection. Evidence to date indicates that blood test trends have been increasingly studied and reported in the context of cancer detection, although knowledge remains fragmented and largely exploratory. Evidence is concentrated on a small number of cancers, particularly colorectal and pancreatic, with substantial gaps for many other cancer types. Broader investigations across a wider range of cancers and blood tests are needed. While most studies focused on the association between blood test trends and cancer or on prediction models incorporating trends for cancer risk estimation, more comprehensive research is required to further explore their role in cancer detection, including their diagnostic value and the perspectives of patients and clinicians on their use in cancer detection.

## Supplementary Information

Below is the link to the electronic supplementary material.

Supplementary Material 1

## Data Availability

The datasets used and/or analysed during the current study are available from the corresponding author on reasonable request.
