# Supplementary material for: The use of blood test trends in cancer detection: a scoping review
Source: Diagn Progn Res. 2026 Sep 14;10:24. doi: 10.1186/s41512-026-00236-9 (PMC13573397; doi:10.1186/s41512-026-00236-9)
Supplement: Supplementary file 1 [file 41512_2026_236_MOESM1_ESM.docx]

**Appendix 1 Example search strategy in MEDLINE (Ovid)**

1 exp Neoplasms/bl [Blood]

2 exp Neoplasms/

3 (neoplas* or tumor* or tumour* or cancer* or malignan* or carcino* or sarcom* or leukaem* or leukem* or lymphom* or melano* or metasta* or mesothelio* or mesotelio* or carcinomatos* or gliom* or glioblastom* or osteosarcom* or blastom* or neuroblastom* or oncolog* or myelodysplas* or adenocarcinoma* or choriocarcinoma*).ti.

4 2 or 3

5 Hematologic Tests/

6 exp Blood Cell Count/

7 exp Blood Cells/an

8 Blood Sedimentation/

9 Blood Viscosity/

10 exp Hemoglobins/an, bl

11 Hematocrit/

12 Erythrocyte Indices/ or Erythrocytes/an, bl

13 platelet function tests/ or mean platelet volume/

14 Liver Function Tests/

15 alanine transaminase/an, bl or exp aspartate aminotransferases/an, bl

16 Albumins/an or Albuminuria/bl, ur

17 serum albumin/ or serum albumin, human/

18 exp Bilirubin/an, bl

19 alpha-Fetoproteins/an, bl

20 Alkaline Phosphatase/an, bl [Analysis, Blood]

21 exp Kidney Function Tests/

22 Sodium/an, bl, ur

23 Potassium/an, bl, ur

24 Creatinine/an, bl, ur

25 Urea/an, bl, ur

26 Amylases/an, bl

27 Calcium/an, bl, ur

28 Glycated Hemoglobin A/an, bl or Blood Glucose/an, bl

29 Blood Proteins/an, bl

30 C-Reactive Protein/an, bl

31 exp Thyrotropin/an, bl

32 ((hemoglobin? or haemoglobin? or hb) adj3 (variation? or level? or concentration? or declin* or mean cell)).ti,ab,kf. or (hemoglobin? or haemoglobin?).ti.

33 ((complete blood or whole blood or blood cell or white cell or erythrocyte? or leukocyte? or platelet? or lymphocyte? or eosinophil? or neutrophil? or basophil? or monocyte?) adj2 (count? or variation?)).ti,ab,kf.

34 ((lymphocyte? or eosinophil? or neutrophil? or basophil? or monocyte?) adj2 (percent* or "%")).ti,ab,kf.

35 (erythrocyte sedimentation or blood sedimentation or blood viscosity or mean platelet or mean cell volume or hematocrit? or haematocrit? or ((blood cell or erythrocyte?) adj2 (index or indices or distribution))).ti,ab,kf.

36 ((c-reactive protein or crp) adj3 (plasma or serum or blood or variation? or level? or concentration? or elevat? or increas* or high*)).ti,ab,kf. or (c-reactive protein or crp).ti.

37 ((total or serum or blood or plasma) adj2 protein?).ti,ab,kf. or protein.ti.

38 ((albumin? adj3 (plasma or serum or blood or variation? or level? or concentration? or declin*)) or albumin creatinine ratio?).ti,ab,kf. or albumin*.ti.

39 ((alkaline phosphatase or alp) adj3 (plasma or serum or blood or variation? or level? or concentration? or elevat? or increas* or high*)).ti,ab,kf. or (alkaline phosphatase or alp).ti.

40 ((aminotransferase or ast or sgot or alt or sgpt) adj3 (plasma or serum or blood or variation? or level? or concentration? or elevat? or increas* or high*)).ti,ab,kf. or (aminotransferase or ast or sgot or alt or sgpt).ti.

41 (bilirubin adj3 (plasma or serum or blood or variation? or level? or concentration? or elevat? or increas* or high*)).ti,ab,kf. or bilirubin.ti.

42 (liver enzyme? adj3 (plasma or serum or blood or variation? or level? or concentration? or elevat? or increas* or high*)).ti,ab,kf. or (liver enzyme? or liver function).ti. or liver function test*.ti,ab,kf.

43 (renal funtion test* or kidney function test*).ti,ab,kf. or (renal function or kidney function).ti.

44 (sodium adj3 (plasma or serum or blood or variation? or level? or concentration? or declin*)).ti,ab,kf. or sodium.ti.

45 (potassium adj3 (plasma or serum or blood or variation? or level? or concentration? or elevat? or increas* or high*)).ti,ab,kf. or potassium.ti.

46 (creatinine adj3 (plasma or serum or blood or variation? or level? or concentration? or elevat? or increas* or high*)).ti,ab,kf. or creatinine.ti.

47 ((urea adj3 (plasma or serum or blood or variation? or level? or concentration? or decline?)) or urea cycle?).ti,ab,kf. or urea.ti.

48 (amylase adj3 (plasma or serum or blood or variation? or level? or concentration? or elevat? or increas* or high*)).ti,ab,kf. or amylase.ti.

49 (((glucose or hba1c) adj3 (plasma or serum or blood or variation? or level? or concentration? or elevat? or increas* or high*)) or fasting glucose).ti,ab,kf. or (glucose or hba1c).ti.

50 (calcium adj3 (plasma or serum or blood or variation? or level? or concentration? or elevat? or increas* or high*)).ti,ab,kf. or calcium.ti. or calcium adjusted.ti,ab,kf.

51 (((thyrotropin? or thyroid stimulating hormone?) adj3 (plasma or serum or blood or variation? or level? or concentration? or elevat? or increas* or high*)) or fasting glucose).ti,ab,kf. or (thyrotropon? or thyroid stimulating hormone?).ti.

52 anemia/ or anemia, hypochromic/ or anemia, iron-deficiency/ or exp anemia, macrocytic/

53 (an?emia? or an?emic or microcytosis or microcytic).ti,ab,kf.

54 or/5-53

55 4 and 54

56 1 or 55

57 exp Neoplasms/di

58 early diagnosis/ or "early detection of cancer"/

59 (detect* or diagnos* or screen*).ti.

60 ((neoplas* or tumor* or tumour* or cancer* or malignan* or carcino* or sarcom* or leukaem* or leukem* or lymphom* or melano* or metasta* or mesothelio* or mesotelio* or carcinomatos* or gliom* or glioblastom* or osteosarcom* or blastom* or neuroblastom* or oncolog* or myelodysplas* or adenocarcinoma* or choriocarcinoma*) adj3 (diagnos* or detect* or screen*)).ti,ab,kf.

61 57 or 58 or 59 or 60

62 56 and 61

63 (trend? or pattern? or lead time* or timeline* or time line* or time frame? or time frame? or interval?).ti,ab,kf.

64 ((longterm or long-term) adj2 (variation? or chang* or difference* or declin* or decreas* or increas* or elevat* or level? or concentration)).ti,ab,kf.

65 (prediagnos* or pre-diagnos* or ((before or prior) adj2 diagnos*)).ti,ab,kf.

66 ((risk? or predict*) adj5 (model* or logarithm* or algorithm* or machine learning)).ti,ab,kf.

67 (model* or logarithm* or algorithm* or maching learning).ti.

68 (risk? adj2 (scor* or model* or index or indices or tool* or assessment? or measurement?)).ti,ab,kf.

69 (Models, Biological/ or Models, Statistical/) and exp Risk/

70 63 or 64 or 65 or 66 or 67 or 68 or 69

71 62 and 70

72 exp animals/ not humans/

73 71 not 72

Appendix 2 Detailed description of included studies

| Study ID | Type of publica-tion | Study design | Location | Study setting | Patient population | Sample size | Number of blood tests per person | Time span for trend | Nature of repeated measurement | Analysis of blood test trends | Findings about blood test trends for cancer detection |
| --- | --- | --- | --- | --- | --- | --- | --- | --- | --- | --- | --- |
| Adiga 2016 | Abstract | Case report | US | Local healthcare centre | 66-year-old Hispanic gentleman with history of hypertension, diabetes mellitus, cerebro vascular accident, intermittent polyarthritis on aspirin and NSAID | 1 | 3 | 3 months | sporadic | Descriptively reported the repeated blood tests | This cancer patient with NHL has consistent low hb, MCV, high ESR and high sensitivity CRP (CRP increased greatly within the last month) |
| Alcala 2023 | Journal article | Cohort | UK | UK Biobank | General population in the UK | 463178 | 1 | Unclear | Regular interval | Strength of association (between blood tests and cancer) over time | Accounting for RCC risk factors attenuated the association between eGFR and RCC risk, but lower eGFR remained associated with higher RCC risk within the last five years  leading up to diagnosis. The association of hazard ratio for lower eGFR with RCC risk gradually attenuated with increasing time from blood draw to diagnosis, and there was no risk increase among participants diagnosed more than five years after recruitment. For instance, the risk factor‐adjusted hazard ratio (HRsd) estimates for one standard deviation decrease in eGFR measured with creatinine were 1.18 (95% confidence interval [95% CI]: 1.13‐1.23) when measured one year prior to diagnosis, 1.08 (95% CI: 1.04‐1.13) five years before diagnosis, and 1.03 (95% CI: 0.98‐1.07) 10 years before diagnosis. |
| Andersen 2017 | Abstract | Cohort | Denmark | Copenhagen Primary Care Laboratory (CopLab) Database | Patients diagnosed with Chronic lymphocytic leukemiain the Greater Copenhagen area between 2008 and 2016 | 710 | Median=7 (IQR 3-13) | 15-16 years; median 8 years (IQR 5-11) | sporadic | Fit regression line to decribe the trends | The estimated onset of lymphocyte progression was 4.98 years (95% CI [5.79, 4.09]) before the diagnosis. The median yearly progression in the lymphocyte count was 31% (IQR [23%, 39%]). |
| Atkin 2020 | Journal article | Cohort | UK | Local healthcare centre | Patients diagnosed with multiple myeloma | 285 | Min=10; max=2535 | 13 years | Sporadic | Statistical tests to compared blood test values at different time points in different groups | There was a statistically significant decline in mean haemoglobin over 2 years prior to diagnosis (p<0.0005). Haematocrit appeared to decline over the same time, but  this was not statistically significant. There appeared to be an increase in mean corrected calcium and creatinine in the 90 days preceding diagnosis, but not before. |
| Ayling 2019 | Journal article | Cohort | UK | Local healthcare centre | Patients seen in the Gastroenterology Clinic in Derriford Hospital, Plymouth, between March 2014 and March 2017, who had been referred from Primary Care with a low haemoglobin concentration, ostensibly secondary to iron deficiency, on a 2-week wait cancer pathway | 592 | Unclear | Unclear | Unclear | Trends represented by evaluating each parameter at 2 time points using linear model; fit trend into machine learning for predicton of cancer risk | Of 592 patients referred on the basis of haemoglobin concentration, 21 (3.5%) had CRC. Using ColonFlag, rather than haemoglobin concentration, in combination with symptoms, would have resulted in prioritisation of 304 patients for urgent referral rather than 592. One CRC would have been missed but might have been detected by FIT, which was not available in this case. In patients aged <55 years in whom the incidence of CRC is low, 15 rather than 109 patients would have been prioritised for urgent referral with no cancers missed. |
| Ayling 2021 | Journal article | Cohort | UK | Local healthcare centre | All adult patients over 40 years of age, referred to Barts Health NHS Trust on an urgent pathway with suspected CRC and awaiting investigation on 1 May 2020 | 532 | Median=8 | Unclear | Unclear | Trends represented by evaluating each parameter at 2 time points using linear model; fit trend into machine learning for predicton of cancer risk | ColonFlag to have equal sensitivity and greater specificity than f- Hb at a cut- off of 10 µg/g as a triage tool for CRC |
| Bakheet 2016 | Journal article | Case report | Malaysia | Local healthcare centre | 71-year-old man, with a background history of diabetes  mellitus, hypertension and chronic kidney disease | 1 | Unclear | Unclear | Unclear | Descriptively reported the repeated blood tests | Patient with IgD multiple meyloma may have deteriorating renal function and decreasing trend of haemoglobin levels |
| Barlow 2022 | Abstract | Cohort | UK | Clinical Practice Research Datalink | Lung cancer patients matched on age, sex, and practice at a ratio of 1:5 to healthy controls | 27796 | Unclear | 500 days | Sporadic | Strength of association (between blood tests and cancer) over time | The platelet count began to rise 300 days before lung cancer diagnosis, with SCC patients experiencing a significantly faster rise than ADC and SCLC patients. |
| Birks 2017 | Journal article | Cohort | UK | Clinical Practice Research Datalink GOLD | Patients had at least one FBC present in the record | 2914589 | 11.8 | Average 11 years | Sporadic | Trends represented by evaluating each parameter at 2 time points using linear model; fit trend into machine learning for predicton of cancer risk | The area under the curve for the 18–24- month interval was 0.776 [95% confidence interval (CI): 0.771, 0.781]. Performance improves as the time interval reduces. The area under the curve for the age- matched case control analysis was 0.583 [0.574, 0.591]. For the population risk- scored in 2012, the positive predictive value at 99.5% specificity was 8.8% with negative predictive value 99.6%. |
| Boursi 2016 | Journal article | Case-control | UK | The Health Improvement Network (THIN) | People receiving medical care from 1995 to 2013 from a THIN practitioner | 67988 | Unclear | 6.3 years | Sporadic | Building prediction model for cancer using change of blood tests over time but the trend was not selected in the final model | A model similar to existing CRC prediction models which included age, sex, height, obesity, ever smoking, alcohol dependence, and previous screening colonoscopy had an AUC of 0.58 (0.57–0.59) with poor goodness of fit. A laboratory-based model including hematocrit, MCV, lymphocytes, and neutrophil–lymphocyte ratio (NLR) had an AUC of 0.76 (0.76–0.77) and a McFadden’s R2 of 0.21 with a NRI of 47.6 %. A combined model including sex, hemoglobin, MCV, white blood cells, platelets, NLR, and oral hypoglycemic use had an AUC of 0.80 (0.79–0.81) with a McFadden’s R2 of 0.27 and a NRI of 60.7 %. |
| Boursi 2022 | Journal article | Cohort | Israel | Maccabi Health Services; Israel Cancer Registry | Patients 50 years or older that had a physician diagnosis of NOD at the MHS diabetes registry | 5408 | Unclear | 18 months | Regular intervals, every 3-18 months | Quantified changes in blood tests (absolute or relative changes) | Performance of cancer prediction model incorporating blood test trends: AUC/C-statistic =0.69 |
| Brewer 2022 | Journal article | Case-control | US | Database complied by the Mount Sinai Data Warehouse | Patients with diagnosis of PDAC and age 45 to 90 years; matched controls | 4626 | Unclear; subgroup analyses excluded patients with less than 2 tests | 5 years | Sporadic | Average value at different time points with signiciant tests between case and control; slope included in predcition model | At Y5 and Y4, there were no differences in HbA1c between cases and controls. At Y3, Y2, Y1, and YDX, a higher median HbA1c was observed in cases compared with controls . Similar trends were observed when comparing glucose values of cases and controls. Multivariable logistic regression demonstrated that HbA1c slope (adjusted odds ratio, 1.33; 95% confidence interval, 1.01-1.76) and BMI slope (adjusted odds ratio, 0.75; 95% confidence interval, 0.65-0.87) were predictors of PDAC after adjusting for race, smoking, alcohol use, sex, and age |
| Capizzi 1992 | Journal article | Case report | US | Mayo Clinic | A 70-year-old man was evaluated for longstanding intermittent jaundice | 1 | 2 | Unclear | Sporadic | Descriptively reported the repeated blood tests | Total serum bilirubin dropped from 8.7 mg/dL before referral to 6.7 after referral |
| Castañeda-Avila 2022 | Journal article | Cohort | US | electronic health records (EHR)  and health claims data for patients receiving care at Reliant Medical Group | Patients aged 50 years and older with Monoclonal gammopathy of undetermined significance MGUS | 424 | Cr tests: median=12 (range: 2–119) and Hgb: median=10 (range: 2–156) | 3 years | Sporadic | Trajectory group included in risk model | After adjusting for Charlson comorbidity index, age at diagnosis, and sex, patients in group 3 (aOR: 5.28, 95% CI = 1.79–15.58) and group 2 (aOR: 12.16; 95% CI  = 1.02–144.79) were more likely to eventually be diagnosed with multiple myeloma than patients in group 1 |
| Chaturvedi 2010 | Journal article | Case-control | US | Local healthcare centre | men and women age 55 to 74 years from 1992 to 2001 | 1262 | Unclear | 6 years | Regular intervals, every 1 year | Strength of association (between blood tests and cancer) over time | The relationship of CRP with lung cancer did not vary significantly by follow-up time,although the association appeared strongest in the period 2 to 5 years before lung cancer diagnosis (Q4 v Q1:OR,2.33; 95%CI,1.24 to 4.39) |
| Chen 2021 | Journal article | Cohort | US | Patient data  from Kaiser Permanente Southern California (KPSC) | Patients between 50 and 84 years of age who met the following criteria for NOD | 13947 | 2 | 1 year | Sporadic | Quantified changes in blood tests (absolute or relative changes) | In the high risk group, the 3-year risk of PDAC and sensitivity in the original END-PAC study were 3.6% and 78%, respectively, higher than the corresponding figures (2.0% and 63%) at KPSC |
| Chen 2024 | Journal article | Cohort | US | Kaiser Permanente Southern California | Patients had an elevated HbA1c | 79060 | Unclear | 36 months | Regular intervals, every 3-18 months | Quantified changes in blood tests (absolute or relative changes) | Performance of cancer prediction model incorporating blood test trends: AUC/C-statistic 0.75 |
| Claridge 2024 | Journal article | Protocol for a cohort | UK | 20 primary care prac tices | At least 50 years old and with new- onset diabetes identified in the last 3 years | NA | At most 6 | 2 years | Sporadic | Quantified changes in blood tests (absolute or relative changes) | NA -protocol; no reults reported |
| Edgren 2010 | Journal article | Case-control | Sweden and Denmark | SCANDAT | Donors for whom at least one successful whole blood, plasma or platelet donation was recorded between the January 1st, 1968 and December 31st, 2002, and who had no history of cancer at the time of the first recorded donation | 178370 | Median=10 | 5 years | Sporadic | Fit regression line to decribe the trends, strength of association (between blood tests and cancer) over time | At baseline, 5 years before the case diagnosis, there were no apparent differences between cases and controls. For cancer, overall there was only a slight hemoglobin concentration decline among the cases as they approached their cancer diagnosis. Five years before the index date only lymphatic leukemia and all sites combined departed significantly from null, but the effects were quite minimal. Further on, however, for stomach cancer, multiple myeloma, and lymphatic leukemia, a decreased hemoglobin concentration was associated with an increased cancer risk starting already 3 years before the diagnosis. For cancer overall, person-specific hemoglobin concentration changes functioned poorly as a screening tool (AUC ¼ 0.53; 95% CI 0.53–0.54) |
| Feng 2020 | Journal article | Cohort | China | Local healthcare centre | Employees from the Kailuan Group older than 18 years including retired individuals from the Kailuan Group with FBG data | 69742 | Unclear | 4 years | Regular intervals, every 2 years | Trajectory group included in risk model | Relative to the moderate-stable group, we found a higher hazard ratio (HR) for overall cancer in the low-increasing group (HR = 1.26, 95%confidence interval (CI) 1.06–1.50); and for gastrointestinal cancer in the elevated-stable group (HR = 1.66, 95%CI1.22–2.26) |
| Fisher 1999 | Thesis | Cohort | US | Data from a RCT (LRCCPPT) and its foloow up | Patients aged 35-50 with type 2 hyperlipoproteinemia | 2333 | Unclear | 8 years | Regular interval every 1 year | Fit regression line to decribe the trends; slope of the regression line | Eight years before cancer and MI diagnoses mean serum albumin is significantly lower than eight years prior to the end of the trial in the group of study participants not diagnosed with cancer or heart disease, evidenced by non-overlapping 95 percent confidence intervals. After adjustment for age at randomization, serum albumin decreases prior to all events - cancer, MI, and, for the “neither” group, prior to end of the trial. However, the decrease is at least marginally statistically significant only for the following groups: all cancers combined, prostate cancer, MI, and “neither”. The steepest slope is seen in the group of study participants developing prostate cancer. |
| de la Fuente 2019 | Journal article | Case-control | US | Rochester Epidemiology Project | Patients who had either biopsy-proven PDAC or a pancreatic mass suspicious for adenocarcinoma with elevated CA19-9 or obstructive jaundice | 657 | Unclear | 2 years | Sporadic | Average value at different time points with signiciant tests between case and control; regression line | Comparing values between the groups 24 months prior to PDAC diagnosis, mean PBM counts (10⁹/L) were similar between cases and controls in the intervals from months 18–24 (0.61 vs. 0.62; p = 0.83), 18–12 (0.62 vs. 0.62; p = 0.91), and 12–6 (0.62 vs. 0.61; p = 0.86).The mean PBM counts were significantly different between cases and controls in the interval from 6 months to 1 month prior to diagnosis (0.69 vs. 0.61; p = 0.03) and continued to rise until PDAC diagnosis (0.73 vs. 0.59; p < 0.001). |
| Furukawa 1984 | Journal article | Cohort | Japan | Unclear | Patients with liver cirrhosis confirmed by laporoscopy and liver biopsy. None of them were treated with steroids. | 114 | Unclear | Unclear | At least once a month | Derived trajectory groups based on blood test temporal patterns | The incidence of LCC was 28% in group A, 19% in groupB and 18% in group C. there is no significant difference among these three group |
| Garg 2011 | Abstract | Case report | US | Local healthcare centre | A previously healthy 71-year-old female | 1 | 3 for albumin and 2 for CRP | 2.5 years | Sporadic | Descriptively reported the repeated blood tests | Albumin: 4.3 in mar 2006, 1.7 in sep 2008 and 1.6 in oct 2008; CRP 0.08 in dec 2007 and 11.0 in oct 2008 |
| Garg 2019 | Journal article | Case-control | US | Mayo Clinic tumor registry | Subjects who had biopsy-proven pancreatic ductal adenocarcinoma and reported a family history of pancreatic cancer in a first-degree relative | 12 | Unclear | 5 years | Sporadic | Average value at different time points with signiciant tests | Mean blood sugar in FPC group 5 years prior to pancreatic cancer diagnosis was lower compared to at diagnosis (102.5 +14.1 mg vs 131.4 +46.9 mg, p = 0.04) |
| Giannakeas 2022 | Thesis | Cohort | Canada | Electronic medical records from the province of Ontario | Patients with cancer diagnosis between January 2007 to December 2015 | 213336 | 2 | 2 years | Sporadic | Fit regression line to decribe the trends | For ovarian cancer patients, there is a slow rise in platelet count for the first 18 months and then a rapid rise in the six months prior to diagnosis, when the  two curves begin to split. At diagnosis, patients who died from ovarian cancer have a much higher median platelet count than the patients who survived . Similar trends were seen for colon cancer, lung cancer, and stomach cancer, for both female and male patients. For breast and prostate cancer, the separation was minimal before diagnosis but not after diagnosis |
| Giannakeas 2024 | Abstract | Cohort | Canada | Provincial electronic health data, from Ontario, Can ada | Adults aged 40 to 75 with two consecutive routine CBC records ordered 9 to 15 months apart by their family doctor | 1331671 | 2 | 15 months | Sporadic | Slope/ratio of change of blood tests | Several variables were strongly associated with an ovarian cancer diagnosis; features that were most predictive include absolute and relative changes in platelet count, lymphocyte count, and neutrophil count. Overall model performance decreased from time from CBC to cancer diagnosis, when looking at follow-up intervals of <1 year, 1 to <3 years, and 3 to 5 years |
| Goldshtein 2010 | Journal article | Case-control | Israel | Maccabi Healthcare Services | MHS members aged 45–75 years who have been diagnosed with CRC between 1/1/2004 and 14/1/2009 | 10740 | Unclear | 10 years | Regular intervals, every 8 months | Average value at different time points with signiciant tests between case and control; regression line | Starting from 4 years prior to cancer diagnosis, a progressive significant (P<0.001) decrement in Hb levels (0.28 g/dl per 6 months) wasfound amongcasesbutnot amongcontrols. CRCpatients were characterized in an on-going, long-term, logarithmic decrement in Hb levels. |
| Goshen 2018 | Journal article | Cohort | Israel | Maccabi Health Maintenance Organization | Individuals who had a CBC result reported within the study period and who were not compliant with standard screening recommendations | 79671 | Unclear | Unclear | Unclear | Trends represented by evaluating each parameter at 2 time points using linear model; fit trend into machine learning for predicton of cancer risk | The ColonFlag identified 688 men and women who scored in the highest 0.87 percentile. Of these individuals, 254 had colonoscopies performed by Maccabi physicians, and 19 CRCs (7.5%) were found. An additional 15 cancers primarily identified outside of Maccabi were found through code matching |
| Gould 2021 | Journal article | Case-control | US | Kaiser Permanente Southern California | Case patients included KPSC members with lung cancer diagnosed between 2008 and 2015 who were 45–90 years old on the date of diagnosis. Control subjects included KPSC members without a current or prior diagnosis of lung cancer who were 45–90 years old on an assigned index (pseudodiagnosis) date of July 1 | 117669 | Unclear | 5 years | Sporadic | Machine learning incorporating the dynamic properties of blood tests | Among ever-smokers in the test set, a machine learning model was more accurate than the mPLCOm2012 for identifying non-small cell lung cancer (NSCLC) 9–12 months before clinical diagnosis (P < 0.00001). The model demonstrated an area under the curve (AUC) of 0.86, a diagnostic odds ratio (OR) of 12.3, and a sensitivity of 40.1% at a predefined specificity of 95%. The machine learning model was more accurate than standard eligibility criteria for lung cancer screening and also outperformed the mPLCOm2012 when applied to a screening-eligible population. |
| Gradel 2020 | Journal article | Cohort | Denmark | Danish National Acute Leukemia Registry; Danish National Patient Registry; Danish Civil Registration System; biochemistry laboratory information systems; OUH Patient Administrative System; microbiology laboratory information system | All patients with AML who were followed at the Department of Haematology, Odense University Hospital (OUH) | 818 | Unclear | 30 days | Unclear | Average value at different time points in cases | CRP levels showed no clear trend of increase or decrease during the 30 days leading up to the diagnosis date. Most mean CRP levels ranged from 55 to 95 mg/L during this period. There were no conspicuous changes in mean CRP levels at the time of AML diagnosis, but a minor increase began approximately 7 days thereafter, continuing until day 20, after which levels declined again. In the 30 days leading up to the diagnosis date, mean PA levels fluctuated around 35 g/L, with no clear trend of increase or decrease. |
| Hauser 2021 | Journal article | Cohort | US | Veterans Health Administration | Patients with a BCR-ABL1 test during the study period | 1623 | Unclear | 6+ years | Sporadic | Developed prediction model using repeatedly measured tests taken at different time points | The predictive ability of machine learning models improved when trained with blood cell counts closer to time of diagnosis: 2 to 5 years area under the curve (AUC), 0.59 to 0.67, 0.5 to 1 years AUC, 0.75 to 0.80, at diagnosis AUC, 0.87 to 0.92. |
| Hilsden 2018 | Journal article | Cohort | Canada | Alberta Health Services’ Analytics, Alberta Cancer Registry | Individuals between the ages of 50 and 75 who underwent a colonoscopy at the Centre between January 2013 and June 2015 | 17676 | Unclear | Unclear | Sporadic | Trends represented by evaluating each parameter at 2 time points using linear model; fit trend into machine learning for predicton of cancer risk | Among the 17,676 individuals who underwent a screening colonoscopy there were 1,014 found to have a high risk precancerous lesion (5.7%) and 60 were found to have colorectal cancer (0.3%). At aspecificity of 95%, the odds ratio for a positive ColonFlag was 2.0 for those with an advanced precancerous lesion compared with those with a normal colonoscopy. |
| Holt 2023 | Journal article | Case control and cohort | UK | Clinical Practice Research Datalink GOLD | Patients aged of 40 years or above with at least one FBC in their primary care | 1893641 | At least 1 | 30 months | Sporadic | Trends represented by evaluating each parameter at 2 time points using linear model; fit trend into machine learning for predicton of cancer risk | Symptoms appear relatively late in the colorectal cancer process and are limited for supporting early stage detection. The ColonFlag can discriminate usefully at 18–24 months before diagnosis, suggesting a role for this algorithm in primary care, although some of its discriminatory ability comes from the age variable. |
| Hornbrook 2017 | Journal article | Case-control | US | Kaiser Permanente Northwest Region; Kaiser Permanente Tumor Registry | The colorectal cancer cases were patients from the KP Tumor Registry who had one or more CBCs within 6 months of the CRC diagnosis date. Control cases were patients who received at least one outpatient CBC with no history of cancer diagnoses | 17095 | Unclear | Unclear | Sporadic | Trends represented by evaluating each parameter at 2 time points using linear model; fit trend into machine learning for predicton of cancer risk | Area under the receiver operating characteristics curve for detecting colorectal cancer was 0.80 ± 0.01. At 99% specificity, the odds ratio for association of a high-risk detection score with colorectal cancer was 34.7 (95% CI 28.9–40.4). |
| Hsieh 2019 | Journal article | Cohort | US | Local healthcare centre | Patients who had at least 2 readings of high platelet counts within 30 days of each other seen at Mayo Clinic | 305 | Unclear | 30 days | Unclear | Descriptively reported the repeated blood tests | For outpatients, hematologic malignancy (56.9%), asplenia (36.7%), and iron deficiency (28.4%) were the most common causes. Hematologic malignancy was 3.4 times more likely to be the cause of EXT in outpatients than in inpatients (56.9% vs. 16.8%), and a new hematologic malignancy was 1.9 times more likely to be diagnosed in outpatients (15.6% vs. 8.2%) |
| Huang 2020 | Journal article | Cohort | US | Kaiser Permanente Southern California | Patients with 45–90 years of age, 2 years of continuous membership in KPSC system, and available information for body mass index (BMI), smoking, and alcohol use | 110699 | Unclear | 3 years | Unclear | Average value at different time points without significant test; slope of change | Cases and noncases had similar values and minor increases in fasting glucose in the 3 years to 1 month before diabetes diagnosis. However, the change in slope immediately before diabetes diagnosis was much higher for cases compared with noncases (cases: 37.47 mg/dL; noncases: 27.68 mg/dL; P for heterogeneity < .01) . This pattern was similar for HbA1c, in which cases and noncases had similar values and trends up to 1 month prior (cases: 0.03%/interval; noncases: 0.04%/ interval; P for heterogeneity =.38), but cases had a much larger change in slope compared with noncases during the interval right before diabetes (cases: 1.39%; non cases: 0.86%; P for heterogeneity < .001). For HbA1c, whites and blacks had the largest differences between cases and noncases for the change in slope. |
| Iversen 1996 | Journal article | Case-control | Norway | Norwegien Cancer Registry | RCC patients in Norway are reported to the Nowegien Cancer Registry; with control matched | 4146 | Unclear | 6 years | Sporadic | Average value at different time points without significant tests; regression line | An on-the-average steady increase became demonstratable in both sexes, with a markedly sharp elevation during the last year before the malignant condition became apparent. The mean ESR value were consistently and significently higher in women than in men. The avaerage increase during the consecutive 6-1 year before diagnosis was 2.3 in men and 3,0 in women |
| Jacobson 2021 | Journal article | Case-control | Sweden | Swedish cancer registry | NSHDS participants that were diagnosed with pancreatic cancer in the Swedish cancer registry. Controls without any cancer diagnosis were selected from the NSHDS | 899 | Unclear | Unclear | Unclear | Strength of association over time; average value at different time points without significant tests, regression line | Over time, fasting glucose increased more steeply in cases compared with matched controls in a linear regression model adjusted for age (P for interaction = 0.009). In addition, mean fasting glucose levels were significantly higher in cases at both 0–3 and 3–6 years prior to cancer diagnosis compared with controls. We observed a higher overall odds ratio (OR) for developing pancreatic cancer with each mmol/L increase in fasting glucose level (OR 1.30, 95% CI 1.05–1.60, P = 0.02). The association between fasting glucose and pancreatic cancer was stratified by the lag time between sampling and diagnosis. The odds ratio for a lag time of 3–6 years before diagnosis was significantly increased (OR 1.49, 95% CI 1.05–2.10, P = 0.02, n = 39), while the odds ratio for 0–3 years before diagnosis approached significance (OR 1.47, 95% CI 0.99–2.19, P = 0.06, n = 27). |
| Jonsson 2020 | Journal article | Case-control | Sweden | Swedish Cancer Register | Incident cases were diagnosed with glioma after the sampling. Controls (cancer-free at inclusion) with single or repeated samples were randomly selected | 264 | Unclear | 11.7 years | Sporadic | Derived trajectory groups based on blood test temporal patterns | For glioma, we detect significant changes in metabolite levels as early as eight years before diagnosis, with longitudinal follow up within seven years. Elevated blood plasma levels of myo-inositol, cysteine, N-acetylglucosamine, creatinine, glycine, proline, erythronic-, 4-hydroxyphenylacetic-, uric-, and aceturic acid were particularly evident in glioma cases. |
| Kawamura 2011 | Abstract | Cohort | Japan | Local healthcare centre | Patients with Non-alcoholic fatty liver disease(NAFLD) diagnosed by ultrasonography | 6508 | Unclear | Median=5.6 years | Unclear | Descriptively reported the repeated blood tests | The number/porportion of cancer patients with a specific trend in platelet count |
| Khan 2021 | Journal article | Cohort | US | TrinetX | Patients were new-onset diabetes | 6301 | Unclear | 1 year | Unclear | Quantified changes in blood tests (absolute or relative changes) | Performance of cancer prediction model incorporating blood test trends: AUC/C-statistic 0.72 |
| Kim 2017 | Abstract | Cohort | Korea | Unclear | Treatment-naive, chronic hepatitis B patients who started entecavir therapy between January 2007 and June 2012 | 894 | 2 | 1 year | Unclear | Derived trajectory groups based on blood test temporal patterns; slope | The cumulative incidence rate at 5-year was significantly different according to the dynamic status of ALBI grade (7.9%, 7.7%, 17.4% and 26.7% for group 1 (n = 511), group 2 (n = 229), group 3 (n = 18), and group 4 (n = 136), respectively). |
| Kinar 2016 | Journal article | Cohort | Israel/UK | Maccabi Healthcare Services; Israeli National Cancer Registry/The Health Improvement Network | All insured individuals aged 50-75 years | 466107 | Unclear | 3 years | Regular intervals, every 18 months | Trends represented by evaluating each parameter at 2 time points using linear model; fit trend into machine learning for predicton of cancer risk | Using blood counts obtained 3–6 months before diagnosis, the area under the curve for detecting CRC was 0.8260.01 for the Israeli valida tion set. The specificity was 88.62% in the Israeli validation set and 94.61% in the UK dataset. |
| Kinar 2017 | Journal article | Cohort | Israel | Maccabi Health Services; Israel Cancer Registry | Patients between ages 50 and 75 on January 1, 2008 who had one or more CBC report recorded in the MHS electronic medical record system for a blood sample taken during the six month testing period | 112584 | Unclear | Unclear | Unclear | Trends represented by evaluating each parameter at 2 time points using linear model; fit trend into machine learning for predicton of cancer risk | For individuals in the highest one percentile, the odds ratio for CRC was 21.8 (95%CI 13.8to34.2). For the majority of the individuals with cancer, CRCwasnotsuspectedatthetimeof theblood draw. Frequent use of anticoagulants, the presence of other gastrointestinal pathologies and non-GI malignancies were assocaitged with false positive MeScores. |
| Klatte 2024 | Journal article | Cohort | US | Mayo Clinic  Cancer Registry | 18 years of age with a diagnosis of PDAC who had received at least 1 of 10 commonly used blood-based biomarker tests in the 36 months before diagnosis | 3306 | At least 1 | 3 years | Sporadic | Regression line; quantified changes in blood tests (absolute or relative changes) | On a group level, fasting blood glucose increased prior to diagnosis, although the occurrence of hyperglycaemia was less clearly reflected in haemoglobin A1c. The other biomarkers (albumin, C-reactive protein, haemoglobin, and white blood cells) showed no distinct changes approaching PDAC diagnosis. The observed trends were consistent across both early and late disease stages. |
| Koshiaris 2018 | Journal article | Case-control | UK | Clinical Practice Research Datalink | Cases were patients were >40 years of age with a myeloma diagnosis | 14860 | Unclear | 5 years | Sporadic | Average value at different time points without significant test | In patients with myeloma, but not in controls, haemoglobin levels began to decrease approximately 3 years prior to diagnosis in both males and females. The mean haemoglobin value met the WHO criteria for anaemia throughout the final 24 months in males (13 g/dL) and the final 15 months in females (12 g/dL). Mean corpuscular volume began to increase approximately 1.5 years before diagnosis, although the rise was modest. White cell count (WCC) and platelet levels remained stable and within the normal range at all time points. Mean calcium and creatinine levels rose rapidly during the final 3–6 months prior to diagnosis. Erythrocyte sedimentation rate (ESR) and plasma viscosity (PV) began to increase approximately 2 years before diagnosis. Importantly, C-reactive protein (CRP) showed no difference between cases and controls at any time point. |
| Kubo 2016 | Journal article | Case report | Japan | Printing company | Patients with occupational cholangiocarcinoma | 9 | Unclear | 12 years | Regular intervals, every year | Descriptively reported the repeated blood tests | In all nine patients, serum γ-GTP activity was elevated several years before the detection of cholangiocarcinoma. Serum ALT activity also increased several years prior to diagnosis, typically following an earlier rise in serum AST activity in most patients. |
| Lemanska 2022 | Journal article | Case-control | UK | ORCHID | Patients with pancreatic cancer diagnosis, or any of the listed above pancreatic cancer features at the age of 40+ years | 43756 | Unclear | 6 years | Sporadic | Strength of association (between blood tests and cancer) over time; regression line | Although HbA1c increased prior to the diagnosis of pancreatic cancer in both subgroups, the change began approximately 2 years earlier (−3 years vs. −1 year) and was greater in cases with diabetes compared with those without diabetes. The increase in HbA1c was statistically significant from 3 years before pancreatic cancer diagnosis. Odds ratios (ORs) decreased and statistical significance diminished beyond 3 years before diagnosis in individuals with diabetes, and beyond 1 year before diagnosis in those without diabetes. |
| Li 2021 | Journal article | Case-control | Sweden | Swedish Colorectal Cancer Registry | All patients who had at least one hemoglobin test requested in primary care during 2000–2017, Aug 22 and who were above the age of 40 at the time of testing were identified | 16867 | Mean: Cases=3; controls=2 | 16.5 years | Sporadic | Fit regression line to decribe the trends | In controls, haemoglobin concentration remained relatively stable for up to 16.5 years prior to the hypothetical CRC diagnosis, with no non-linear temporal trend observed in either sex. In contrast, CRC patients demonstrated a statistically significant non-linear temporal trend in haemoglobin concentration in both sexes, with a sharp decline approaching diagnosis. Cut-off analysis indicated significantly lower haemoglobin concentrations 609 days (20 months) prior to CRC diagnosis in men and 905 days (30 months) in women, compared with controls. |
| Mathewson 2023 | Abstract | Case report | US | Outpatient laboratory | A 70-year-old male with a history of type 2 diabetes mellitus | 1 | More than 3 | 1 year | Regular interval | Descriptively reported the repeated blood tests | HbA1c measurements over the past year demonstrated values ≥6.5% with a downward trend, consistent with the diagnosis and treatment of diabetes mellitus.An atypical HbA1c measurement was subsequently identified, in which capillary electrophoresis (CE) analysis flagged an abnormal electropherogram profile and no HbA1c value was reported. This abnormal finding prompted further investigation, including complete blood count (CBC) and oncology testing, ultimately leading to a cancer diagnosis. |
| McCaughanI 2009 | Abstract | Cohort | UK | CausewayHospital,Coleraine | Patients diagnosed with colorectal cancer | 194 | 186 patients with at least one haemoglobin test | 2 years | Sporadic | Descriptively reported the repeated blood tests | A total of 186 patients had a previous haemoglobin measurement available. Of these, 76 patients (41%) were anaemic prior to diagnosis, and a further 25 (13%) experienced a ≥10% decrease in haemoglobin from baseline. Among these patients, 18% had a low mean corpuscular volume (MCV), while no patients had an elevated MCV. Of the 101 patients with either anaemia or a ≥10% haemoglobin drop, 37 (37%) underwent iron studies. Ferritin levels were <50 mg/L in 73% of cases and <100 mg/L in 97%. A normal MCV was observed in 64% of patients with ferritin <100 mg/L. |
| Mueller 2019 | Journal article | Case-control | UK | Clinical Practice Research Datalink | Cases were patients with pancreatic cancer between January 2004 and December 2013 and incident DM within 2 years before the cancer diagnosis  For each pancreatic cancer case, up to 10 controls at random from among CPRD subjects who had new-onset DM and no diagnosis of pancreatic cancer | 6074 | No blood glucose(case=241, control=1998); blood glucose at single time interval (case=174 control=1381); blood glucose in at least two time intervals (case: lower level=107; higher level=66; control: lower level =33; higher level=1174) | 5 years | Sporadic | Strength of association (between blood tests and cancer) over time | Lower blood glucose levels were associated with a greater risk of pancreatic cancer than higher blood glucose levels across the time intervals studied prior to the onset of diabetes mellitus (DM)). In the 2–3 years before the index date, the adjusted odds ratio (aOR) for blood glucose levels ≤5.1 mmol/L (lowest category) was 2.42 (95% CI 1.60–3.66), compared with ≥6.3 mmol/L (highest category). The association between blood glucose levels ≤5.1 mmol/L and pancreatic cancer remained broadly consistent across the pre-diagnostic period, except for a lower aOR of 1.47 (95% CI 0.82–2.64) observed 5–6 years before the index date. |
| Murtola 2018 | Journal article | Cohort | finland | Finnish Randomized  Study of Screening for Prostate Cancer (FinRSPC) linked to the  Fimlab laboratory database to obtain information on fasting plasma/  blood glucose measurements | Men with at least one available measurement of blood glucose and/or HbA1c during 1978–2015 | 17860 | Unclear | Median 14.7 years | Sporadic | Strength of association (between blood tests and cancer) over time; Derived trajectory groups based on blood test temporal patterns | Role of changes in fasting glucose levels after initiation of antidiabetic therapy: Changes in fasting blood glucose levels following the initiation of antidiabetic medication were not associated with changes in prostate cancer risk. Similarly, no association with prostate cancer risk was observed for changes in fasting glucose levels after the initiation of metformin use. Long-term effects of fasting glucose levels: Blood glucose levels measured 10 years earlier were borderline significantly associated with an increased risk of prostate cancer (PCa), consistent with the main analysis. Diabetic-range fasting glucose levels were associated with an increased risk of non-metastatic tumours and tumours with a Gleason score of 6 or less. |
| Ng 2017 | Abstract | Case-control | Hong Kong | Unclear electronic records | Individuals above 40 years of age diagnosed with CRC and additional controls with no record of cancer | 3000 | At least 1 | 8 years | Unclear | Trends represented by evaluating each parameter at 2 time points using linear model; fit trend into machine learning for predicton of cancer risk | Sensitivity was calculated to be 35.6% at a false positive rate of 5% (success criterion was pre-defined to be ≥ 35%) representing a relative risk of 12.5. The model’s AUC (ages 50–75) for CBC test results available up to 6 months prior to the cancer detection, and 6–12 months prior to detection was similar to 2 other previously studied populations (UK and Israel (IL)) (0.78-HK, 0.79-UK, 0.82-IL, and 0.76-HK, 0.75-UK, 0.77-IL, accordingly). Good performance of model was documented for curable CRC stages |
| Oba 2019 | Abstract | Cohort | US | National Cancer Institute’s IRB-approved longitudinal cohort screening study (NCT01443468, http://lfs.cancer.gov) between 2012 and October 2018 | Participants with Li-Fraumeni syndrome (LFS); of the National Cancer Institute’s IRB-approved longitudinal cohort screening study between 2012 and October 2018 | 139 | Median= 9 (range 1-18) | 72 months; median=35 months (range 3-72) | Regular interval, every 4 months | Descriptively reported the repeated blood tests | Of the 139 patients, 33 (24%) developed 45 screen-detected or interval cancers during their study participation. Of the 45 cancers detected, only one (2%), a colorectal cancer, was detected with screening bloodwork due to a down-trending hemoglobin that led to colonoscopy. No hematological malignancies, adrenocortical carcinomas or germ-cell tumors were detected as a result of blood screening. One female participant was diagnosed with polycythemia vera after identification of persistent thrombocytosis. |
| Pannala 2009 | Journal article | Case-control | US | Mayo Diagnostic Index | Patients who had at least one outpatient FBG value in the preceding 60 months or within one month following the index date (provided they received no treatment for pancreatic cancer) | 2611 | Unclear | 5 years | Sporadic | Average value at different time points with signiciant tests between case and control (with regression line) | Mean FBG values were similar between cases and controls in the −60 to −48 (102 vs. 100 mg/ dl, p=0.34) and −48 to −36 month intervals (106 vs. 102 mg/dl, p=0.09) but mean FBG values were progressively higher for cases compared to controls in the −36 to −24 (105 vs. 100 mg/ dl, p=0.01), −24 to −12 (114 vs. 102 mg/dl, p=0.001), and −12 to +1 (123 vs. 102 mg/dl) |
| Putri 2024 | Journal article | review | Indonesia | NA | NA | NA | NA | NA | NA | Review literature on cancer prediction model which used machine learning to analyse blood test trends | Ten studies were identified as eligible. ColonFlag demonstrated area under the curve (AUC) values ranging from 0.736 to 0.82. Sensitivity ranged from 3.91% to 35.4%, while specificity ranged from 82.73% to 94%. Positive predictive values (PPV) ranged from 2.6% to 9.1%, and negative predictive values (NPV) ranged from 97.6% to 99.9%. ColonFlag performed better over shorter time windows, in proximal tumour locations, in more advanced disease stages, and for colorectal cancer (CRC) compared with adenoma. |
| Read 2023 | Journal article | Cohort | US | Local healthcare centre | Individuals from the Michigan Medicine Clinical Data Warehouse who had at least 2 CBCs within a rolling 2-year time frame between 1 January 2004 and 31 December 2013 | 148158 | 2+ | 3 years | Sporadic | Used machine learning to build cancer risk prediction model incorporating blood test trends | For 3-year prediction of GI tract cancers, the longitudinal random forest model performed the best, with an area under the receiver operator curve (AuROC) of 0.750 (95% CI 0.729–0.771) and Brier score of 0.116, compared to the longitudinal logistic regression model, with an AuROC of 0.735 (95% CI 0.713–0.757) and Brier score of 0.205. |
| Rinaldi 2014 | Journal article | Case-control | Europe | Regional cancer registries | Patients who were healthy and aged 35 to 69 years when recruited between 1992 and 1998 in 23 centers in 10 European countries | 1124 | Unclear | More than 11 years | Unclear | Average value at different time points with signiciant tests between case and control | Among case patients, TSH levels were stable over the years before TC diagnosis (P trend= 0.57) |
| Rotkopf 2024 | Letter to editor | Cohort | Israel | Electronic medical data of Rabin Medical Center | Patients with any kind of malignant neoplasm, excluding BCC and SCC of the skin | 4793 | 2 | Unclear | Sporadic | Average value at different time points with signiciant tests | The average HbA1c% levels one year (± 3 months) before diagnosis and at diagnosis (± 3 months) were 7.03± 1.3 % (4 %−16 %) and 6.87± 1.2 % (3.9 %−14.6 %), respectively, a correspondingly difference of 0.15 % [p < 0.001]. |
| Ryan 2023 | Abstract | Case report | US | Local healthcare centre | A 61-year-old male with a history of well-controlled type 2 diabetes mellitus for the past ten years on metformin, glimepiride, and insulin glargine | 1 | 2 | 18 months | Unclear | Descriptively reported the repeated blood tests | Endocrinopathies and gastrointestinal malignancies can lead to uncontrolled HbA1C |
| Sadr-Azodi 2015 | Journal article | Case-control | Sweden | Swedish National Diabetes Register; The Swedish Prescribed Drug Register; The Swedish Cancer Register; The Swedish Patient Register; The Cause of Death Register; The National Education Register; The Register of the Total Population | All patients with diabetes mellitus in Sweden with a record in the Swedish National Diabetes Register (NDR) [14] and who had a dispensed prescription of anti-diabetic drugs | 4301 | Unclear | More than 5 years | Sporadic | Strength of association (between blood tests and cancer) over time | An increase in the risk of pancreatic cancer persisted when comparing the highest to the lowest quartile of HbA1c within the first five years from the clinical detection of pancreatic cancer (p-value for trend 0.05). This association increased almost five-fold within six months from clinical detection of pancreatic cancer (OR 4.73; 95% CI 1.83 – 12.21). This association declined but was increased up to five years before the clinical detection of pancreatic cancer (OR 2.70; 95% CI 0.99 – 7.36 for HbA1c measured 6 months – 1 year before the clinical detection of pan creatic cancer; OR 2.51; 95% CI 1.17 – 5.40 for HbA1c measured 1 – 2 years before the clinical detection of pancreatic cancer; and OR 1.77; 95% CI 0.99 – 5.40 for HbA1c measured 2 – 5 years before the clinical detection of pancreatic cancer). No clear association was found between the timing of HbA1c when comparing the second and third quartile of HbA1c to the lowest quartile. HbA1c measured 5 years before the clinical detection of pancreatic cancer was not associated with this cancer. |
| Sah 2019 | Journal article | Case-control | US | Rochester Epidemiology  Project (REP), a unique medical records linkage system funded by NIH, data from all health care providers in Olmsted County,  Minnesota | PDAC cases in Olmsted County between 2000 and 2015; age- (same birth year) and gender-matched Olmsted County residents as controls | 876 | Unclear | 66 months | Sporadic | Average value at different time points with signiciant tests between case and control; fit regression line to decribe the trends | The mean FBG was similar in cases and controls up to 30 months before index date; it was higher in PDAC vs. controls (p≤0·05) starting 30 to 18-months before index date and progressively increased until diagnosis |
| Sala 2023 | Journal article | Cohort | Spain | Record of La Ribera Health  Department of the Valencian Community | All patients with a diagnosis of CRC, including its ICD10 (International Classification of Diseases) codes for colorectal malignancies | 1878 | Unclear | 450 days | Sporadic | Average value at different time points with signiciant tests between case and control; fit regression line to decribe the trends | A higher incidence of anemia(p < 0.001) was observed among CRLM patients in the three months prior to CRC diagnosis than in CRCpatients showing no LM. A statistically significant deterioration of CBC was observed in patients with advanced-stage CRC and synchronous or early LM (CRLM) in the three months prior to diagnosis. |
| San Tan 2023 | Journal article | Case-control | UK | Qresearch; NCRAS; HES; ONS Death | Adult patients aged 18 years and above | 289356 | Unclear | 5 years | Sporadic | Strength of association (between blood tests and cancer) over time; fit regression line to decribe the trends | The results supported evidence of at least a biphasic increase in HbA1c among pancreatic ductal adenocarcinoma (PDAC) cases, but not in controls. This differential biphasic pattern was most evident in individuals with long-standing diabetes and those without type 2 diabetes (T2D). It began with an early, gradual increase starting approximately 33 months prior to the index month, followed by a late, rapid increase beginning around 18 months prior. In patients with recent-onset T2D, HbA1c levels increased beginning approximately 42–45 months prior to the index month, followed by a gradual decline in both cases and controls, likely reflecting diagnosis of T2D and subsequent glycaemic management. However, in cases with recent-onset T2D, HbA1c levels began to rise rapidly again approximately 21 months prior to the index month, in contrast to the continued decline observed in controls. |
| Sanchez-Salcedo 2016 | Journal article | Case-control | Spain | Pamplona  International  Early  Lung  Cancer  Action  Program  (P-IELCAP | Participants in the P-IELCAP programme between September 2000 and January 2015, who are asymptomatic men and women 40 years of age or older, who are current or former smokers with a tobacco history of at least 10 pack-years | 135 | At least three | 8 years | Regular interval, every year | Fit regression line to decribe the trends; quantified changes in blood tests (absolute or relative changes) | There were no significant differences between baseline NLR and PLR values between groups (NLR: 2.20 ± 1.10 vs. 2.22 ± 0.89; PLR: 109 ± 32 vs. 120 ± 44, in the lung cancer and control group, respectively). During the study follow-up, the mean annual change in NLR was almost ten times greater in the group that developed lung cancer when compared to the control group (2.56% ± 10 vs. 0.27% ± 6; p = 0.25). Similarly, the mean annual PLR relative change was 11 times greater in the cancer group than in the control group (3.75% ± 9 vs. 0.33% ± 6; p = 0.053) |
| Sato 2012 | Journal article | case study | Japan | Local healthcare centre | Pancreatic cancer cases that underwent blood sugar control (diabetes before the diagnosis of pancreatic cancer, and simultaneous cases diagnosed with both pancreatic cancer and diabetes) | 39 | Unclear | 1 year | Sporadic | Descriptively reported the repeated blood tests | HbA1c wosen and this might trigger diagnosis of cancer |
| Sato 2016 | Abstract | case study | Japan | Department of Endocrinology and Metabolism, Hirosaki University or Hirosaki Medical Institution | Pancreatic cancer cases with DM who were admitted to our department between 1999 and 2015 | 52 | Unclear | Unclear | Sporadic | Slope/ratio of change of blood tests | Cases with DM preceding pancreatic cancer had experienced body weight loss and had a mean increase of 2.0% in HbA1C levels,1 year before they were diagnosed with pancreatic cancer |
| Schneider 2018 | Journal article | Case-control | UK | Clinical Practice Research Datalink | All patients in the CPRD aged 18–89 years with a Read-coded CRC diagnosis between 1 January 2008 and 31 December 2012 | 18476 | Unclear | More than 3 years | Sporadic | Strength of association (between blood tests and cancer) over time | The presence of low levels (compared with normal levels) of all assessed markers within 180 days prior to colorectal cancer (CRC) diagnosis was predictive of CRC. The odds ratio (OR) for haemoglobin (Hb) levels <9 g/dL was 74.25 (95% CI 34.69–158.91); for mean corpuscular volume (MCV) ≤80 fL, 13.94 (95% CI 10.31–18.85); for serum ferritin (SF) <20 ng/mL, 10.66 (95% CI 6.88–16.51); and for red blood cell (RBC) counts <3.5 × 10¹²/L, 4.33 (95% CI 3.15–5.96), compared with normal levels of the respective parameters. Although associations were strongest at extreme values, less pronounced abnormalities (e.g., Hb 10.0–10.9 or 11.0–11.9 g/dL) were also significantly associated with CRC diagnosis. |
| Schneider 2020 | Journal article | Case-control | US | Kaiser Permanente Northwest Region | KPNC Health Plan members between 1996 and 2015 who had at least 1 outpatient CBC test for any indication with at least 3 years of prior CBC data | 308721 | Unclear | Unclear | Sporadic | Trends represented by evaluating each parameter at 2 time points using linear model; fit trend into machine learning for predicton of cancer risk | A high score from the algorithm identified patients with a CRC diagnosis within the next 6 months with 35.4% sensitivity (95% CI, 33.8–36.7) and an AUROC of 0.78 (95% CI, 0.77–0.78). |
| Schwartz 2022 | Abstract | Case-control | US | patients’ Comprehensive Metabolic Panels (CMPs) | Cases were women with epithelial ovarian cancer. Controls are women without a diagnosis of cancer- | 124 | Unclear | Unclear | Sporadic | Slope/ratio of change | The probability of cancer for a 0.04 mg/dL/year increase in a-asc increased with age until age 70 and showed a significant dose-response.The Odd Ratio (OR) of ovarian cancer for a 65 yr old woman with a 0.06 mg/dL increase/year was w3.0; the OR for a 1 mg/dl increase/year was 10. This effect was also seen for early stage tumors and persisted after age adjustment. |
| Sharma 2018 a | Abstract | Case-control | US | Rochester Epidemiology Project (REP) database in patients in Olmsted  County, MN | All PDAC patients between January 2000 and January 2015 in Olmsted County, MN | 65654 | Unclear | 24 months | Sporadic | Average value at different time points with signiciant tests between case and control | Compared to controls, PBM counts are significantly higher in PDAC patients at diagnosis and PBM elevation precedes cancer diagnosis by 6 months. Comparison of PBM between cases and controls at different time points (18 to 24 (0.61 vs 0.62; P =0.95), 18 to12 (0.62 vs 0.62; P = 0.91) and 12 to 6 (0.62 vs 0.61; P = 0.74), but significantly different in the interval from 6 months to 1 month prior to diagnosis (0.69 vs 0.61; P =0.03); Mean PBM counts (109/liter) in PDAC patients at diagnosis were higher than controls (0.73 vs 0.59; P < 0.001) |
| Sharma 2018 b | Journal article | Case-control | US | Mayo Clinic; Rochester Epidemiology Project | All PDAC subjects in Olmsted County between 2000 and 2015 | 659 | Mean=3.5 | 5 years | Sporadic | Average value at different time points with signiciant tests between case and control; regression line | FG increases at 4 years before diagnosis, with an increasing rate of change. Remains steady in controls(The mean FBG was similar in patients and controls in the 60 to 54, 54 to  48, 48 to 42, and 42 to 36-month intervals before the index date. Relative hyperglycemia (mean FBG in patients higher than mean FBG in controls, P .05)wasseenstarting 36 to 30 months before the index date (THG) and progressively worsened in the subsequent time intervals until diagnosis (TDX). FBG in the PDAC cohort peaked above diabetes level (> 126 mg/dL) (onset of diabetes) 6 months before TDX |
| Sharma 2018 c | Journal article | Cohort | US | Rochester Epidemiology Project | All new-onset diabetes subjects in Olmsted County between January 1st, 2000 to December 31st, 2015 | 256 | Unclear | 18 months | On at diagnosis, one 3-18 months beforehand | Quantified changes in blood tests (absolute or relative changes) | In the discovery cohort the END-PAC model identified patients who developed pancreatic cancer within 3 years of onset of diabetes with an area under the receiver operating  characteristic curve value of 0.87; a score of ≥3 identified patients who developed pancreatic cancer with 80% sensitivity and specificity. In the validation cohort, a score of ≥3 identified 7/9 patients with pancreatic cancer (78%), with 85% specificity; the prevalence of pancreatic cancer in subjects with score of ≥3 (3.6%) was 4.4-fold more than in patients with new-onset diabetes. |
| Shaukat 2017 | Journal article | Case report | US | Local healthcare centre | 13-month boy with an incidental finding of thrombocytopenia on routine blood work | 1 | 10 | 10 months | Regular interval, every month | Descriptively reported the repeated blood tests | Patient's WBC count remained in the normal range throughout his follow-up; hemoglobin count over several months, which remained normal as well; monthly complete blood counts continued to show evidence of fluctuating thrombocytopenia, as low as 50,000/L. at diagnosis, hemoglobin of 9.6 g/dL (abnormally low), WBC of 30,000/L (abnormally high), and platelet of 20,000/L (abnormally low) |
| Shukla 2021 | Abstract | Cohort | US | Unclear | Acute Myeloid Leukemia pt diagnosed with and without CHF were selected for analysis | 152 | Unclear | 12 months | Unclear | Average value at different time points with signiciant tests between case and control | At 12 m prior AML diagnosis, EF<50% detected differential expression for WBC [p=0.07], Hemoglobin [p=0.002], platelets [p=0.05] and absolute lymphocyte count (ALC) [p=0.006]. Similarly, EF<50% at 6 m prior AML diagnosis detected differential expression for platelets [p=0.01], ALC [p=0.04] . In contrast, cdCHF detected only differential hemoglobin at 12 m [p=0.01]. |
| Song 2024 | Journal article | Cohort | UK | uk biobank | Adults registered in the UK National Health Service (NHS) ages 40 to 69 years | 443540 | Unclear | 8 years | Sporadic | Fit regression line to decribe the trends | For lung cancer, total WBC and neutrophil count were elevated for cases compared with the population average, regardless of the time to cancer diagnosis; similar patterns were seen for cancers of the breast, and kidney, although the elevations appeared smaller. In contrast, CLL/SLL showed a higher total WBC count than the population mean, which was much higher when cases were diagnosed within the first 4 years of follow-up (i.e., within 5 years of the baseline blood draw); this pattern was driven by the high lymphocyte counts, and the NLRs were correspondingly low |
| Stroud 2020 | Journal article | Cohort | US | Local healthcare centre | Participants aged 18+ years who underwent a primary bariatric surgery | 2107 | Unclear | 12 months | Regular intervals, every 1 year | Slope/ratio of change | Reduced cancer risk was observed in percent decrease from baseline for glucose (per 10%, HR= 0.94, 95 %CI 0.96-0.99) |
| Suarez 2020 | Abstract | Case report | US | Local healthcare centre | 86 year old female with jaundice and scleral icterus | 1 | At least 3 | 1 year | Unclear | Descriptively reported the repeated blood tests | HbA1C 1 year prior was 6.7 and HbA1C during the admission was 8.5. During the month preceding her pancreatic cancer diagnosis, her family noticed her blood  glucose levels were running higher than usual. |
| Tarawneh 2024 | Journal article | Cohort | Jordan | Dataset from various hospitals in Jordan | Patient with and without cancer in various hospitals in Jordan | 9222 | Unclear | Unclear | Unclear | Repeated measurements were included in cancer risk prediction model separately | The importance of features were shown: WBC_min being the most important, followed by WBC_CV, PLT_std, etc |
| Tateishi 2021 | Journal article | Case-control | Japan | Records from diabetes clinics of 81  teaching hospitals | Patients with type 2 diabetes mellitus (T2DM) who were initially diagnosed with HCC at participating hospitals between 2010 and 2015, were negative for  both hepatitis B surface antigen (HBsAg) and anti-HCV antibody, and had been followed up at a diabetes clinic at the same hospital for at least 5 years before the diagnosis of HCC | 3516 | 2 | 5 years | Sporadic | Average value at different time points with signiciant tests between case and control | Compared with values 5 years prior to diagnosis, several laboratory parameters showed significant changes at the time of diagnosis. Fasting glucose decreased from 143 (121–181) mg/dL to 132 (110–173) mg/dL (p = 0.02), and HbA1c decreased from 7.1% (6.5–8.1) to 6.9% (6.3–7.6) (p < 0.0001). Albumin levels declined from 4.07 ± 0.41 g/dL to 3.83 ± 0.57 g/dL (p < 0.0001), while haemoglobin decreased from 13.9 ± 1.8 g/dL to 12.7 ± 2.2 g/dL (p < 0.0001). Creatinine increased from 0.80 (0.68–0.98) mg/dL to 0.87 (0.71–1.13) mg/dL (p < 0.0001); etc. |
| Toriola 2011 | Journal article | Case-control | Finland | Finnish Maternity Cohort; Finnish Cancer Registry | Pregnant women at the municipal maternity care units | 340 | Unclear | 15 years | Two samples at least 1 year apart | Derived trajectory groups based on blood test temporal patterns | Women whose CRP concentrations increased over time by >100% of the initial value had an almost two-fold significant increased risk of ovarian cancer, OR 1.90 (95% CI 1.12–3.23, P value 0.03), compared with those with minimal changes in CRP concentrations. Exclusion of subjects with extreme values did not affect the risk estimates.  Compared to people with minimal change, people who had sustaintial decrease in crp didn't have significant difference in OR of cancer OR1.56(0.71-3.45) |
| Toriola 2013 | Journal article | Case-control | US | WHI-OS | Women who were postmenopausal, 50–79 years old and unlikely to relocate or die within 3 years | 1976 | Unclear | 3 years | Two samples 3 years apart | Slope/ratio of change | The largest quintile increase in CRP concentration from baseline to year-3 follow-up (>73%)was associatedwith a non significant increased risk of CRC(OR=1.34, 0.91–1.95,  p-trend=0.13).No tumor-site-specificdifferences were observed. |
| Virdee 2022 a | Journal article | Case-control | UK | Clinical Practice Research Datalink; NCRAS | Patients aged 40+ years with at least one FBC blood test within 10 years before index date were included | 939949 | Unclear | 10 years; male cases=3.0 years; male controls=2.7; female cases=3.3; female controls=2.8 | Sporadic | Fit regression line to decribe the trends; individual trajectory | Trends in most FBC tests were associated with colorectal cancer |
| Virdee 2022 b | Journal article | Cohort | UK | Clinical Practice Research Datalink; NCRAS | Patients aged at least 40 years with at least one haemoglobin, mean corpuscular volume (MCV), and platelet measurement available in their primary care record were included | 497411 | Male: Development: cases=3, non-cases=2; validation: cases=3, non-cases=2; Female: Development: cases=3, non-cases=3; validation: cases=4, non-cases=3 | 5 years; median=2.4 years | Sporadic | Derived individual trajectory of blood tests | Performance of cancer prediction model incorporating blood test trends: male: Brier score 0.0034; AUC/C-statistic0.739 (95% CI=0.726–0.753); female : Brier score 0.0027; AUC/C-statistic0.753 (95% CI=0.737–0.769) |
| Wang 2024 | Journal article | Cohort | US | Veterans Health Administration (VHA), which is the largest integrated healthcare system in the United States and captures inpatient, outpatient, and emergency department encounters | 40 years old hospitalized for acute pancreatitis | 51613 | 2 | 1 year | Sporadic | Repeated measurements were included in cancer risk prediction model separately | Positive laboratory predictors on admission included log(alkaline phosphatase), log(ALT), and log(bilirubin). Negative laboratory predictors included log(alkaline phosphatase) prior to admission and log(AST) on admission. LDL was a weakly positive predictor, while HDL was a weakly negative predictor for 0-2 month cancer;  Our secondary model predicted PC diagnosed between 3 months to 2 years after AP diagnosis. The strongest positive predictors included being a current smoker and log(alkaline phosphatase) on admission. Negative predictors included log(alkaline phosphatase) prior to admission, and log(triglycerides). Again, LDL was a weakly positive predictor, while HDL was a weakly negative predictor. |
| Wu 2024 | Journal article | Cohort | US | Research Data Warehouse of the Kaiser Permanente Southern California (KPSC) | 50–85 years of age who had at least one clinic-based visit | 1844643 | Unclear | 5 years | Unclear | Included in the change of blood tests in prediction model but the trend was not selected in the final model | Trend information was not selected in the final prediction model |
| Zanders 2016 | Journal article | Cohort | Netherlands | Eindhoven Cancer Registry  (ECR) linked on a patient level to the PHARMO Database  Network | Colon cancer (CC) and rectal cancer (RC) patients registered in the ECR-PHARMO cohort between1January1998 and 31 December 2011 | 438 | 5.5±3.0 for colon cancer patients ; 5.4±2.5 for rectal cancer patients | 2 years | Sporadic | Average value at different time points without significant test; quantified changes in blood tests (absolute or relative changes) | HbA1c levels over the 24 months prior to diagnosis (measured at 3-month intervals) showed a relatively stable trend from −24 to −12 months, followed by a decreasing trend from −12 months to diagnosis. Patients with rectal cancer consistently had higher HbA1c values compared with those with colon cancer throughout the entire period. Colon cancer: In a linear model, the intercept (representing HbA1c at diagnosis) was 6.9% (51.6 mmol/mol; Table 2). The full model demonstrated that mean HbA1c decreased by 0.12% per year (95% CI 0.06–0.18; 1.3 mmol/mol; p = 0.0002) in the period prior to diagnosis. Rectal cancer: In a crude linear model, the intercept (HbA1c at diagnosis) was 7.1% (53.5 mmol/mol; Table 2). Prior to diagnosis, mean HbA1c decreased by 0.18% per year (95% CI 0.08–0.28; 2.0 mmol/mol; p = 0.0006). |
| Zhen 2025 | Journal article | Cohort | China | Single-center retrospective cohort with a hospital in Dalian City, China | Patients aged between 20 and 85 years with chronic hepatitis B | 4843 | Unclear | 9 years; median=4.22 years | Unclear | Average value at different time points without significant test; derived individual trajectory of blood tests | To ensure the model’s normality assumption, this study applied a logarithmic transformation to APR, yielding ratio. Ratio levels were higher in females (t = 5.26, P < 0.01). A 1-unit increase in ratio correlated with a 2.005-fold higher risk of HCC in males (95%CI: 1.653-2.431) and a 2.273-fold higher risk in females (95%CI: 1.620-3.190). Baseline ratio levels are not associated with HCC risk, whereas the longitudinal increase in the ratio elevates HCC risk. |
